# Understanding the evolutionary structural variability and target specificity of tick salivary Kunitz peptides using next generation transcriptome data

**DOI:** 10.1186/1471-2148-14-4

**Published:** 2014-01-07

**Authors:** Alexandra Schwarz, Alejandro Cabezas-Cruz, Jan Kopecký, James J Valdés

**Affiliations:** 1 Institute of Parasitology, Biology Centre of the Academy of Sciences of the Czech Republic, 37005 České Budějovice Czech Republic

**Keywords:** Red Queen hypothesis, Tick saliva, Kunitz-domain proteins, Cysteine motif, Structural bioinformatics, Antigenicity, Protein disorder, Molecular clock, Evolution

## Abstract

**Background:**

Ticks are blood-sucking arthropods and a primary function of tick salivary proteins is to counteract the host’s immune response. Tick salivary Kunitz-domain proteins perform multiple functions within the feeding lesion and have been classified as venoms; thereby, constituting them as one of the important elements in the *arms race* with the host. The two main mechanisms advocated to explain the functional heterogeneity of tick salivary Kunitz-domain proteins are gene sharing and gene duplication. Both do not, however, elucidate the evolution of the Kunitz family in ticks from a structural dynamic point of view. The Red Queen hypothesis offers a fruitful theoretical framework to give a dynamic explanation for host-parasite interactions. Using the recent salivary gland *Ixodes ricinus* transcriptome we analyze, for the first time, single Kunitz-domain encoding transcripts by means of computational, structural bioinformatics and phylogenetic approaches to improve our understanding of the structural evolution of this important multigenic protein family.

**Results:**

Organizing the *I. ricinus* single Kunitz-domain peptides based on their cysteine motif allowed us to specify a putative target and to relate this target specificity to Illumina transcript reads during tick feeding. We observe that several of these Kunitz peptide groups vary in their translated amino acid sequence, secondary structure, antigenicity, and intrinsic disorder, and that the majority of these groups are subject to a purifying (negative) selection. We finalize by describing the evolution and emergence of these Kunitz peptides. The overall interpretation of our analyses discloses a rapidly emerging Kunitz group with a distinct disulfide bond pattern from the *I. ricinus* salivary gland transcriptome.

**Conclusions:**

We propose a model to explain the structural and functional evolution of tick salivary Kunitz peptides that we call *target-oriented evolution*. Our study reveals that combining analytical approaches (transcriptomes, computational, bioinformatics and phylogenetics) improves our understanding of the biological functions of important salivary gland mediators during tick feeding.

## Background

Ticks have been successful as ectoparasites due to morphological and physiological adaptations [1]. As obligate hematophagous (blood-sucking) arthropods, ticks transmit various bacterial and viral diseases, e.g., babesiosis, theileriosis, anaplasmosis, Lyme disease and tick-borne encephalitis, and, thus greatly impact human and animal health [2]. To combat host defense mechanisms, ticks possess powerful pharmacological proteins in their saliva that are injected into the vertebrate host during blood feeding [3,4]. Our current understanding of host-parasite interactions has been greatly impacted by the revolution in sequencing technologies that has provided a massive amount of data previously unimaginable [5]. Among the various transcriptome studies to date, transcriptome projects from arthropod disease vectors are using Sanger or next generation sequencing techniques. These include several salivary gland (SG) transcriptome and proteome projects (also known as sialomes) from hard tick [6-18] and soft tick species [19-22]. Hundreds of novel SG transcripts that putatively encode proteins were discovered from these sialome projects, thus elucidating how ticks may complete a blood meal while providing insight towards their evolutionary expansion [23].

Available SG transcriptomes show that the most frequently secreted tick salivary protein families are lipocalins, enzymes, and protease inhibitors. Among protease inhibitors, Kunitz-domain transcripts are one of the most abundant protein families in tick SGs [9,18,19,21]. The archetypal Kunitz fold is highly conserved, resembling the first Kunitz-domain protein, the bovine pancreatic trypsin inhibitor (BPTI) that was functionally described in 1936 by Moses Kunitz [24]. To date, about 15 single Kunitz-domain peptides from ticks have been functionally characterized. Classical serine protease inhibitors were analyzed from the hard ticks *Rhipicephalus microplus* (BMCL) [25], *Rhipicephalus appendiculatus* (TdPI) [26], *Rhipicephalus haemaphysaloides* (Rhipilin-1) [27]*, Haemaphysalis longicornis* (HlChl, HIMKI and Haemangin) [28-30], *Amblyomma cajennense* (Amblyomin-X) [31], and *Ixodes scapularis* (Tryptogalinin) [32]. Protease inhibitors were also characterized from the soft ticks *Ornithodoros moubata* (TAP) [33] and *Ornithodoros savignyi* (FXaI) that mainly function as anti-clotting agents. Anti-platelet inhibitors were also identified as single Kunitz-domain inhibitors, such as the Monogrins (1A and 1B) from *Argas monolakensis*[34], and, their orthologs Disagregin [35] and Savignygrin from *Ornithodoros* spp. [36]. Several tick salivary Kunitz-domain proteins that possess multiple domains (1–7 Kunitz-domains) were also characterized as serine protease inhibitors [37-39]. Of all the tick SGs Kunitz-domain proteins, however, single Kunitz-domain peptides are highly represented (we will refer to these single domains as Kunitz peptides, henceforth) [9,12]. These Kunitz peptides vary in their cysteine (Cys) motifs (possessing more or less than 6 Cys residues) with some lacking the archetypal disulfide bonds causing a more flexible fold, therefore diversifying their inhibitory activity [26,32,40].

Kunitz SG peptides of *I. scapularis* were phylogenetically analyzed to uncover their emergence in ticks and the expression trends of these Kunitz peptides were also statistically analyzed [23]. These Kunitz peptides were categorized in three different groups (groups I, II and III) based on their Cys motif. The *I. scapularis* Kunitz peptides belonging to group I were suggested to represent the ancestor of all tick Kunitz-domain family (single and multiple domains). Several Kunitz peptides seemed to have lost their ability to function as serine protease inhibitors and instead to block and/or modulate ion channels, possibly related to the tick’s necessity for prolonged feeding on the vertebrate host [23]. The authors are aware of only one study that functionally and structurally characterized a tick Kunitz peptide as an ion channel effector, the maxiK channel modulator Ra-KLP from *R. appendiculatus*[40]. This paucity must be remedied since Dai et al. [23] putatively characterize the majority of *I. scapularis* Kunitz peptides as ion channel blockers/modulators. Furthermore, Fry et al. [41] have argued that hematophagous secreted proteins, such as of the Kunitz family, should be classified as venomous. Most classified toxins are stabilized by their disulfide bridges and once these toxins become functionally essential as a venom, their adaptation is often reinforced by gene duplication [41]. Gene sharing and gene duplication are the main mechanisms advocated to explain the functional heterogeneity of tick salivary Kunitz family proteins [23,42].

In our study, we used computational, structural bioinformatics and phylogenetic methods to reevaluate tick salivary Kunitz peptides from a more in-depth structural point of view by analyzing the functional, antigenic, and evolutionary characteristics of Kunitz peptides from the recently annotated *Ixodes ricinus* SG transcriptome [18]; GenBank Bioproject PRJNA177622. Compared to classical biochemical analyses and classical Sanger sequencing techniques that revealed only a few thousands of sequences from tick transcriptome studies presented until today (with the exception of the *A. maculatum* transcriptome), the large amount of available data of the 454 pyrosequencing/Illumina *I. ricinus* SG transcriptome makes it feasible to thoroughly analyze multigenic protein families (i.e., the Kunitz-domain family). In the *I. ricinus* transcriptome, 4% of all Illumina reads were classified as transcripts encoding for Kunitz-domain proteins and of these, 1.4% accounted for single Kunitz-domain transcripts (that putatively encode archetypal Kunitz peptides possessing more or less 6 Cys residues) [18]. Our approach demonstrates how to interpret next generation transcriptome data to expand our understanding of the molecular, structural and functional evolution of hematophagy in ticks. Our overall analysis developed a novel concept that elucidates the divergence and adaptation of *I. ricinus* salivary Kunitz peptides that we call *target-oriented evolution*.

## Results and discussion

### Organizing the different *I. ricinus* SG Kunitz peptides based on their Cys motifs facilitates functional predictions

**Figure 1:**
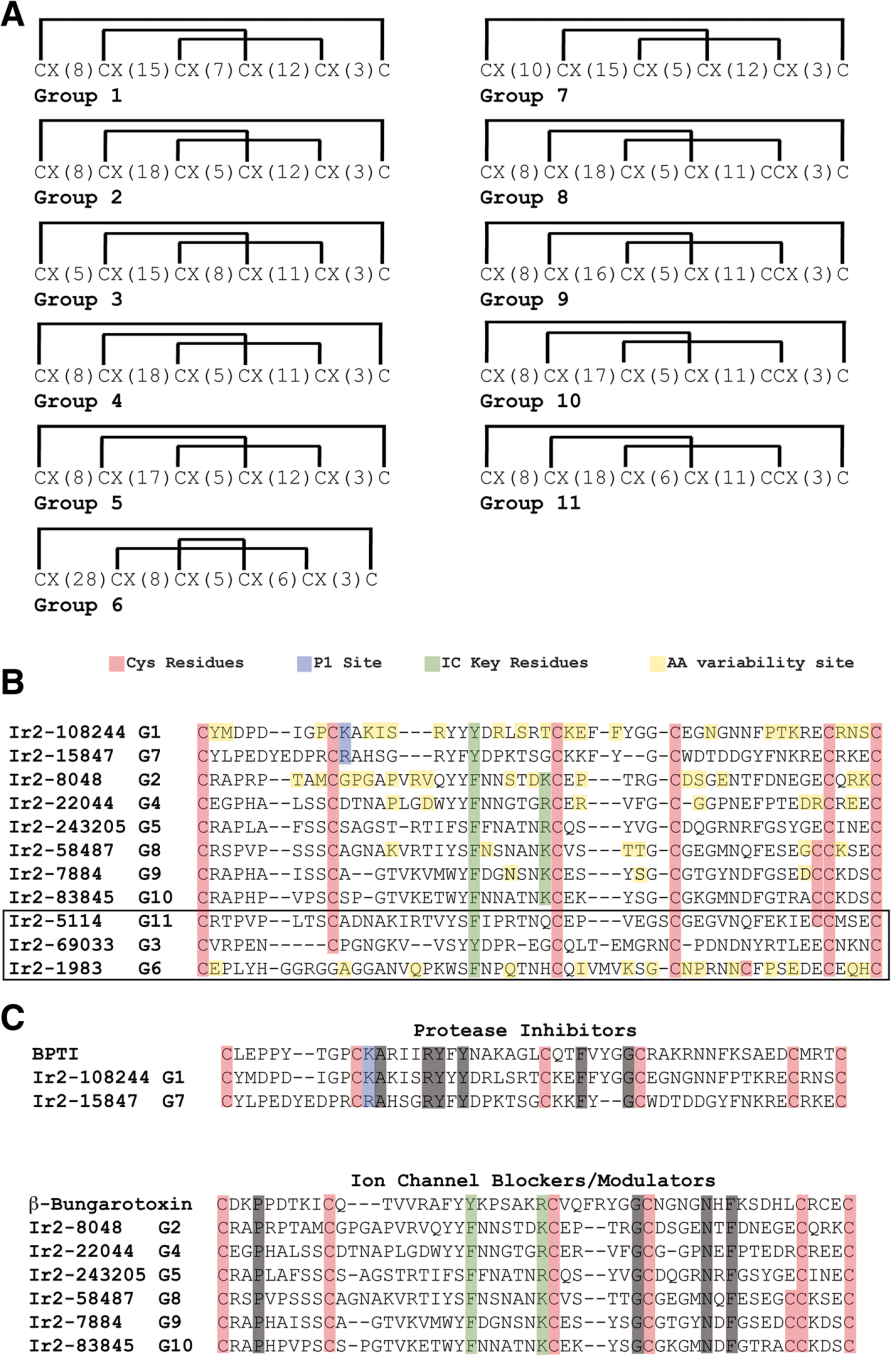
***I. ricinus *Kunitz groups based on Cys motif.** Kunitz peptides from the annotated transcriptome of *I. ricinus* were organized into 11 groups (G1-G11) based on their Cys motif (C = Cys and X = intra-Cys residues). The Cys motif for each group is depicted in Panel **A** along with their predicted disulfide bond patterns (in brackets). The alignment of one representative for each group **(B)** shows the conserved Cys residues (red), the positions of aa variability (yellow), and the key residues that interact with serine proteases (blue) and ion channels (green). The box around G3, G6 and G11 indicates that they do not contain all of the standard residues for serine protease (blue) or ion channel (green) interactions. Two separate alignments in Panel **C** depict the key conserved residues for serine protease inhibition (blue) using BPTI (UniProt: P00974) and ion channel blockage (green) using beta-bungarotoxin (UniProt: P00989). Residues highlighted in black are conserved.

From the 454/Illumina SG *I. ricinus* transcriptome [18], we found archetypal Kunitz peptides containing 6 Cys residues and variants containing an additional 7^th^ Cys residue adjacent to the conserved 5^th^ Cys residue (Figure 1). Based on their Cys motif we were able to organize these tick salivary Kunitz peptides into 11 groups (G1-G11; Figure 1A) and a group with ≤4 Cys in their amino acid (aa) sequences – we will refer to these peptides as *simple Kunitz* (SK), hereafter. The Pfam server (http://pfam.sanger.ac.uk/) verified that all 11 groups plus the SKs possess the conserved single Kunitz-domain. Group G6 is the only *I. ricinus* Kunitz peptide group that possesses a different disulfide bond pattern since it lacks the archetypal Kunitz/BPTI Cys2 and Cys4 disulfide bridge (Figure 1A). The 3D modeled structures of all group representatives from Figure 1 show that the overall archetypal tertiary Kunitz fold is highly conserved regardless of the deviated disulfide bridge pattern for G6 (Additional file 1A-B). Furthermore, the tertiary Kunitz fold for each group representative does not structurally deviate much from each other (Additional file 1C and Additional file 2). The deviation from the archetypal Cys motif (as in G6), however, has been shown to be functionally flexible [26,32,40].

Aligning a representative of each *I. ricinus* Kunitz peptide group (Figure 1B) in our study shows that specific residues coincide with the putative functions described by Dai et al. [23], classified as serine protease inhibitors or ion channel blockers/modulators. Groups G3, G6 and G11, however, do not contain these conserved residues (boxed in Figure 1B). With the exception of the annotated *I. scapularis* sialome [6,9], both protein BLAST [43] and PSI-BLAST [44] searches do not reveal any known or functionally described single Kunitz-domain peptide with the Cys motif of G3, G6 and G11. The proteins that slightly resemble the Cys motif of G6, and lack the archetypal Cys2 and Cys4 disulfide bridge, are the *I. scapularis* salivary Kunitz proteins Ixolaris [38] and Penthalaris [39], both possessing multiple Kunitz domains, and some members of the Kunitz family found in *R. microplus*[45]. An alignment using the classical Kunitz serine protease inhibitor BPTI (UniProt: P00974) with G1 and G7 shows the conserved positively charged arginine (Arg or R) or lysine (Lys or K), known as the P1 site that interacts with the negatively charged active site of serine proteases (highlighted blue in Figure 1C). Furthermore, the key residues for the ion channel blocker beta-bungarotoxin (UniProt: P00989), from the venomous snake *Bungarus multicinctus*[46], are also conserved in G2, G4, G5 and G8-G10 (highlighted green in Figure 1C).

**Figure 2:**
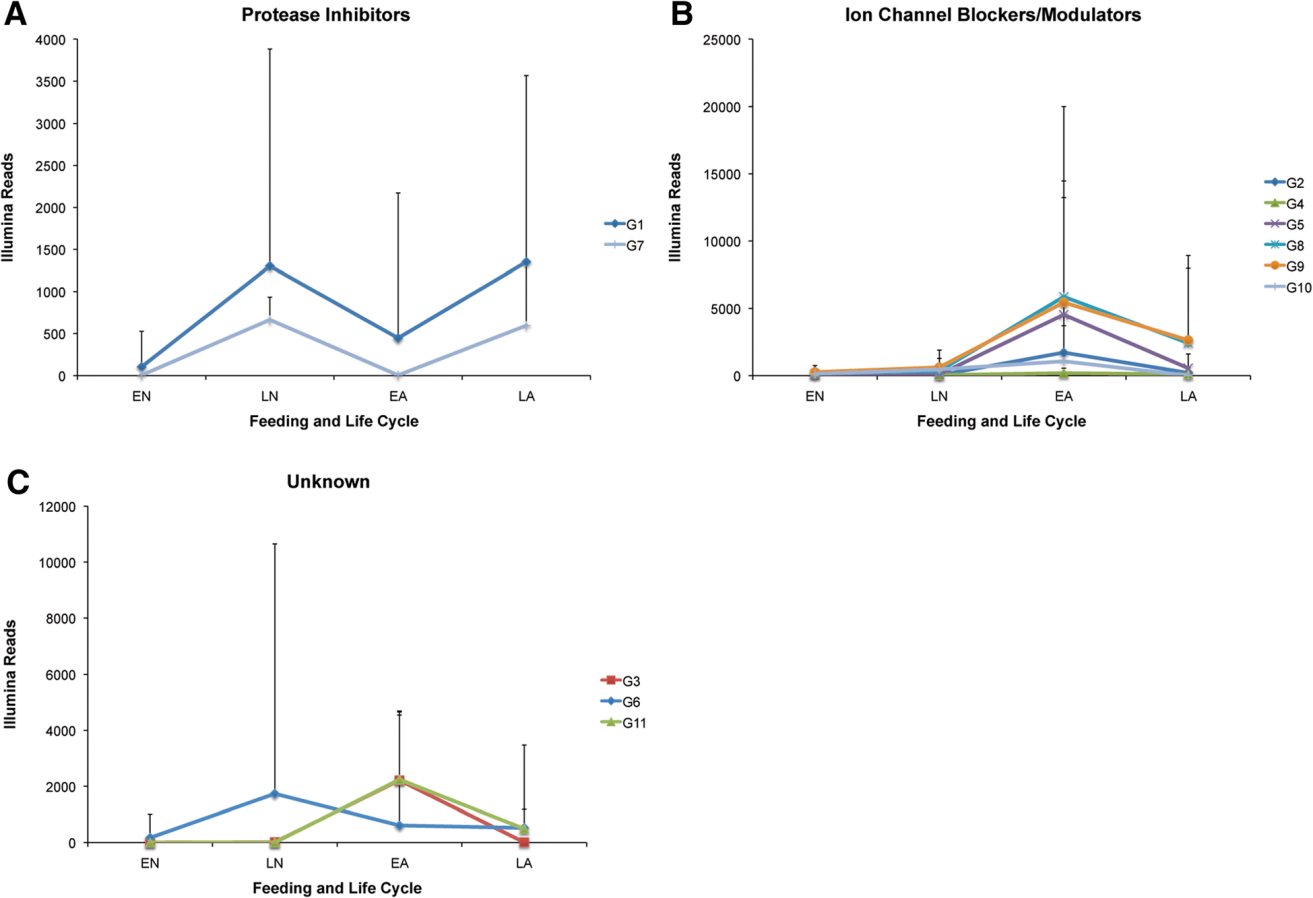
**Average Illumina reads of nymphal and female *I. ricinus *Kunitz-domain transcripts.** Each Cys motif group (G1-G11) was graphed to depict their overall distribution of their average Illumina reads (y-axis) sequenced from four independent *I. ricinus* SG cDNA libraries (x-axis) – two nymphal stages (EN and LN) and two adult stages (EA and LA). For a detailed account on the specifics of these *I. ricinus* cDNA libraries see Schwarz et al. 2013 [18]. Panel **A** represents putative serine protease inhibitors (G1 and G7) with a distinct Illumina read profile in nymphs and female adult SGs of their encoding transcripts compared with the encoding putative ion channel blockers/modulators of panel **B** (G2, G4, G5 and G8-10). Panel **C** depicts that G3 and G11 share a similar profile to ion channel blockers/modulators **(B)** and that Kunitz-domain transcripts of G6 show a third profile in ticks completely distinct from all other groups. Standard deviations are represented as error bars.

By organizing the Kunitz peptides from the *I. ricinus* transcriptome into specific groups, we were also able to visualize specific profiles occurring in the transcript reads among the four independent cDNA libraries, suggesting that there may be a correlation between the average transcript read value and putative function for each group. The three groups with the highest number of transcripts were G1, G2 and G6 (G6 being the largest group). Figures 2A-C depicts the average Illumina reads for all groups obtained from the *I. ricinus* transcriptome, complementing our functional predictions. We notice that three profiles are occurring among the organized transcripts encoding for Kunitz peptides. Figure 2A (G1 and G7) shows that the average transcript read level (Kunitz encoding transcripts that we classify as putative protease inhibitors) is low at the early stages of tick feeding for both nymphs (EN: 6 h to 24 h of feeding on the host) and adults (EA: 6 h to 2 days of feeding on the host) compared with the increased transcript reads during late feeding (LN: 2–4 days of feeding and LA: 3–7 days of feeding). Soft ticks have a shorter feeding cycle than hard ticks and this phenomenon has been thoroughly explained by comparing the evolutionary divergence and emergence of serine protease inhibitors in hard ticks and soft ticks (about 120 and 92 MYA) [42]. Giving that hard ticks (like *I. ricinus*) feed on blood for longer periods of time (females: ≥1 week) and that serine proteases are involved in blood coagulation and immune responses, we expect a higher transcript read encoding for serine protease inhibitors during late feeding stages as depicted in Figure 2A (for both nymphal and adult life cycles).

We putatively classify G2, G4, G5 and G8-G10 as ion channel blockers and/or modulators (Figure 1C) and as Figure 2B shows they exhibit a separate profile than the serine protease inhibitors of Figure 2A (G1 and G7). These putative ion channel blockers/modulators show lower transcript reads during the nymphal stages of feeding (both early, EN, and late, LN), but the reads are increased in adult ticks during the early stage of blood feeding (EA). The profile then decreases during the late stages of feeding (Figure 2B). A possibility, as noted in mayfly nymphs SGs, for the expression of ion channel blockers/modulators in *I. ricinus* nymphs may partially be due to maintaining osmoregulatory function during molting [47]. To resist desiccation, tick nymphs rely on both water vapor and water retention, while adult ticks only rely on enhanced water retention [48]; therefore, this may explain the increased transcript read encoding for ion channel blockers/modulators during adult feeding stages (compared with nymphs – Figure 2B). In Figure 2C we show that G6 deviates from the other two profiles, however, G3 and G11 have similar profiles to that of ion channel blockers/modulators (Figure 2B). The distinct profile and Cys motif G6 displays suggests that this group may belong to (a) a combination of serine protease inhibitors and ion channel blockers/modulators, (b) either serine protease inhibitors or ion channel blockers/modulators, or, (c) a completely new, undefined function – i.e., gene sharing (moonlighting).

### The *I. ricinus* SG Kunitz groups possess intra-Cys residue variability, assemble into various secondary structure clusters, and vary in antigenicity and protein disorder

**Figure 3:**
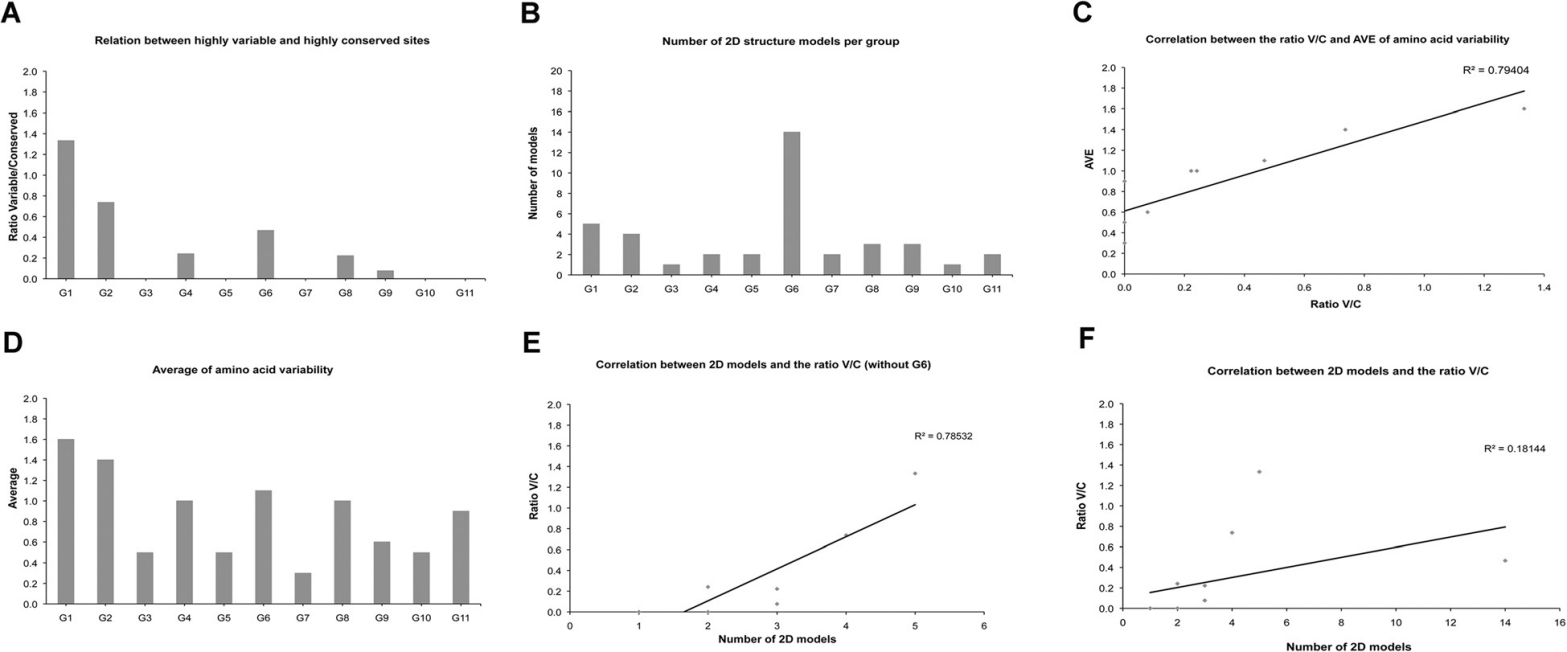
**Graphical representations of amino acid and secondary structure variability.** The relation between highly variable and low variable sites (V/C) among the groups is shown **(A)** The number of secondary structure models per groups is also presented **(B)**. Correlation between the average of aa variability (AVE) and the ratio V/C **(C)**. Average of aa variability estimated by Shannon entropy **(D)** and the correlation between the ratio V/C and 2D models, without and with group 6 **(E-F)**.

The aa positions with high variability (highlighted yellow in Figure 1B) for the intra-Cys residues of each group were calculated by the Protein Variability Server [49,50] using the Shannon entropy equation (see Methods) [51] are represented in Figures 3A and D as the ratio of highly variable/highly conserved sites (V/C) and the average of aa variability (AVE) per group, respectively. On one hand, we consider G3, G5, G7, G10 and G11 are “frozen” groups having low aa variability and high proportion of highly conserved sites. On the other hand, G1, G2 and G6 are “melted” groups or highly variable. Due to their low aa variability, the former assemblage may be considered highly adapted in the host-parasite interaction from a functional and/or immunological point of view. The second assemblage may still be under evolutive pressures that force aa variability.

To demonstrate the structural outcome of these “frozen” and “melted” clusters, we predicted the secondary structure for each Kunitz peptide in all groups. We show that 22 models depict the variability of secondary structure among the 145 Kunitz peptides (excluding SK peptides) (Additional file 3). Five models in Additional file 3 (Representative models *G1: Ir2:98*; *G1: Ir2:4878; G1: Ir2:19262; G6: Ir2:1982* and *G7: Ir2: 4792*) are combined β-strand and α-helical secondary structure models, differing in length, amount and order of α-helices and β-strand, and describe 81.6% of the secondary structure variation present in the *I. ricinus* Kunitz/BPTI family. All the groups contain different amounts of secondary structure models (Figure 3B and Additional file 3). It seems, however, that the secondary structure presented in *Ir2-4878* and *Ir2-98* of G1 is the structural skeleton for the remaining secondary structures; these structures are also present in 10 of the 11 groups and explain 50% of the secondary structures. The fact that G1 represents the structural skeleton may be since its Cys motif (as displayed in Figure 1A) is considered the ancestor of Kunitz peptides in *I. scapularis*[20]. Structural convergence, however, has been reported for venoms, a general classification that the Kunitz protein family belongs to [Revised in 41].

When analyzing the aa variability (V/C or AVE) in relation to the secondary structure models per group (excluding G6) a high correlation is given (R^2^ = 0.78; Figure 3E); however, by including G6 in this analysis the correlation decreases (R^2^ = 0.18; Figure 3F). This dictates that the aa variability per group is partly justifiable for structural reasons (functionality), except in G6. Figure 3C shows that ratio V/C or AVE of aa variability are correlated, therefore, using V/C or AVE of aa variability is interchangeable for Figure 3E and F. The presence of different folds (i.e., secondary structures) suggests that gene sharing events may have occurred throughout the evolution of *I. ricinus* and may cause additional and unique functional properties. Such moonlighting proteins may explain the diverse inhibitory effect of tick salivary Kunitz peptides.

**Figure 4:**
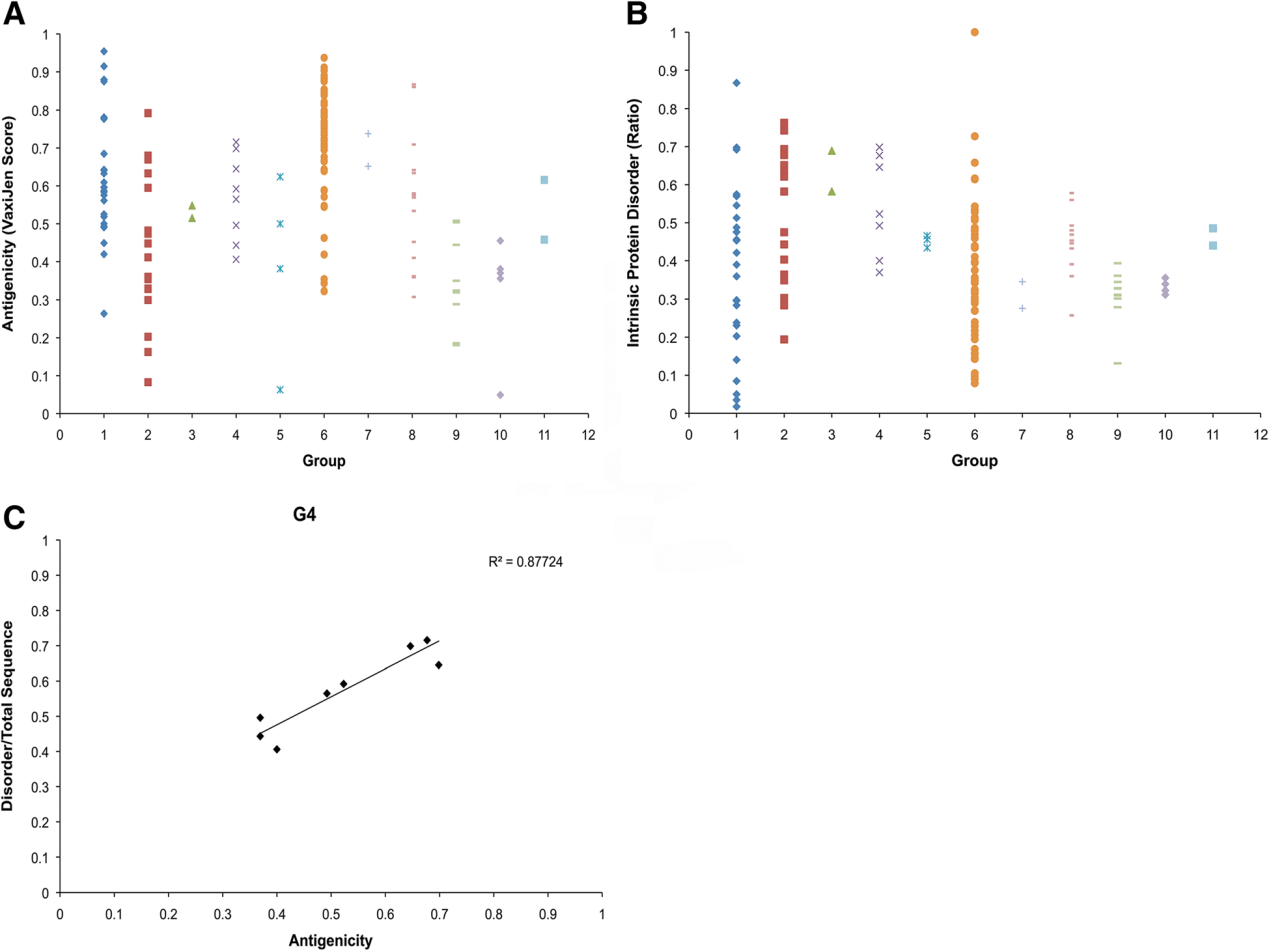
**Antigenicity and intrinsic protein disorder.** Variations in antigenicity **(A)** for each group as predicted by the VaxiJen server [55] that uses an alignment-independent algorithm based on the physicochemical properties of proteins [55]. Ratio of disordered compared to the total sequence **(B)** for each group as predicted by the MetaDisorder server that builds a consensus from 13 protein disorder predictors [56]. Panel **C** shows the correlation for G4 between antigenicity (x-axis) and intrinsic protein disorder (y-axis).

To elucidate additional molecular properties, we analyzed both the antigenic properties and protein disorder for all *I. ricinus* Kunitz peptide groups in our study. Our analysis reveals that the regional disorder was located at both termini for the majority of peptides (data not shown). It is worth noting that antigenic properties involve the mobility of the C– and N-termini [52]. In Figure 4A we show that G6 has the highest antigenicity mean (VaxiJen score). G6 has a “misplaced” Cys2 (archetypal) and lack of this Cys2 is responsible for the functional flexibility of the Kunitz-domain binding loop, that may permit target-specific interactions other than the catalytic domain of serine proteases [53]. We also notice that groups with only two sequences vary in antigenicity (Figure 4A) that also coincides with differences in secondary structure (Additional file 3). Functional flexibility (or mobility) is also demonstrated in G6, since it has a higher variability in intrinsic protein disorder (Figure 4B). Intrinsically disordered regions (or proteins) increase molecular recognition because of an ability to fold differently upon binding and possess large interacting surfaces [54]. Interestingly, G6 has one sequence that is completely disordered (*Ir2-10518*) that is also highly antigenic (>0.7).

### *The I. ricinus* SG Kunitz transcripts are under different selective pressures

The Red Queen hypothesis was proposed by Van Valen in 1973 [57] to explain the molecular evolution through evolutive interactions of prey–predator or host-parasite. The hypothesis establishes *“that a proportional amount of successful response by one species produces a total negative effect on other species[…] To maintain itself as before, the [other] species must increase its fitness [in a proportional amount]”.* The *I. ricinus* SG Kunitz encoding transcripts may adhere to the Red Queen hypothesis, where inverse selection (positive or negative) is a series of one species adapting (the tick) and the other counteradapting (the host). To elucidate or validate the type of selective pressure (positive or purifying) each *I. ricinus* Kunitz peptide group has undergone throughout its evolution we used the Synonymous Non-synonymous Analysis Program (SNAP) (http://www.hiv.lanl.gov) and Datamonkey servers [58] (see Methods for further details).

Assuming that a ratio >1 between the non-synonymous nucleotide substitutions per site (*d*_*N*_) with the synonymous substitutions (*d*_*S*_) is due to a positive selection and a ratio <1 is due to a purifying selection, we note in Table 1 that the majority of groups are under a purifying selection. As we depict in Figure 4C, only G4 had a strong correlation between disorder and antigenicity; coincidentally, G4 is the only group that has undergone positive selection (Table 1). This correlation (Figure 4C) suggests that the disorder in G4 may be under positive selection due to the immune system of the host, since protein mobility (disorder) may influence the antigenic properties of proteins [52]. Our SNAP analysis, however, only displays the average of synonymous and the non-synonymous nucleotide substitutions per site, therefore, is not to say that selection per site was not positive or negative for specific members of the remaining groups. We therefore used Datamonkey to verify our SNAP analyses for evidence of positive selection and to determine which site along the nucleotide sequence are undergoing positive (natural) or purifying (negative) selection.

**Table 1:** Statistical analysis on nucleotide substitution

			**SNAP**					**Datamonkey**		
		***d***_***N***_			***d***_***S***_					
**Group**	**Mean**	**σ**	**Variance**	**Mean**	**σ**	**Variance**	* ***d***_***N***_**/*d***_***S***_	* ***d***_***N***_**/*d***_***S***_	**Evidence	***Selection type
1	0.56	0.02	0.006	1.01	0.18	0.03	0.56	0.69	No	Negative
2	0.34	0.06	0.003	0.56	0.07	0.005	0.61	1.03	Yes	Positive/negative
4	0.25	0.03	0.001	0.17	0.03	0.001	1.47	1.75	Yes	Positive
5	0.31	0.21	0.045	0.88	0.37	0.14	0.35	0.62	No	None
6	0.28	0.04	0.001	0.36	0.04	0.002	0.78	0.94	Yes	Positive/negative
8	0.39	0.08	0.006	0.7	0.09	0.008	0.56	0.72	No	Negative
9	0.17	0.03	0.001	0.34	0.05	0.002	0.5	0.51	No	Negative
10	0.15	0.03	0.001	0.35	0.07	0.005	0.43	0.53	No	None

*The ratio between non-synonymous (*d*_*N*_) and synonymous (*d*_*S*_) nucleotide substitution per site analyzed by SNAP and by Datamonkey via SLAC [59].

**Evidence for positive selection (p-value < 0.05) analyzed by Datamonkey via PARRIS [60].

***Evidence for selection type for specific sites (p-value < 0.05) analyzed via SLAC.

**Figure 5:**
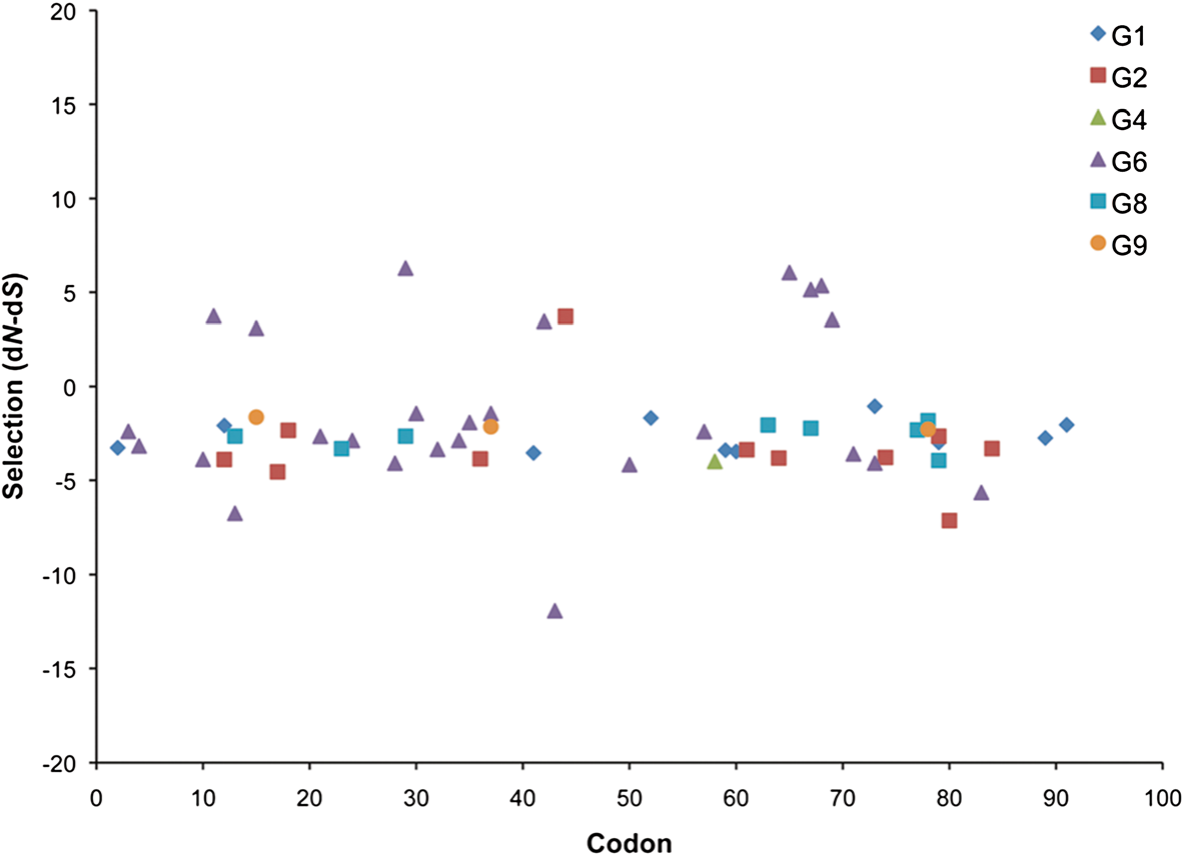
**Positively and negatively selected sites of *I. ricinus *Kunitz transcripts.** Codon-by-codon (x-axis) of the nucleotide substitution selected sites (positive or negative; y-axis) were calculated by the Datamonkey server [58] via the SLAC algorithm [59]. The graph depicts the groups that had evidence (*p-value < 0.05*) for selection as indicated in Table 1. Of note, G3, G7 and G11 (and simple Kunitz) were not included since they contained only two sequences.

Overall, the ratio *d*_*N*_/*d*_*S*_ from Datamonkey slightly differ from that of SNAP. The Datamonkey analyses show that G2, G4 and G6 possess evidence for positive selection and note the type of selection for each site, using the SLAC algorithm (see Methods). Figure 5 graphically displays these results along the entire transcript, codon-by-codon (x-axis). Both G5 and G10 do not possess any sites under any selective pressure, while the remaining groups possess positive, negative (purifying) or both (see Table 1). As depicted in Figure 5, the majority of selected sites are negative (purifying) and groups G2 and G6 possess a few positively selected sites; however, there are more negatively selected sites for G2 and G6 than positively selected sites. In a recently published book, Eugene Koonin [61] explains that purifying (negative) selection, in some phases of evolution, is more common (orders of magnitude more common) than positive selection. Koonin considers purifying selection is the default process of elimination of the unfit. With this regard we understand that Kunitz-domain proteins – taking into account the number of members in general – should undergo a massive purifying selection in order to shape (constrain) the molecular diversity of this tick SG peptide family. Additionally, as noted by the reduced gene duplications events in platypus venom compared with the massive gene expansions reported in cone snails and spiders [62], selective pressures may fluctuate according to venom function.

### The evolution of *I. ricinus* SG Kunitz peptide groups

**Figure 6:**
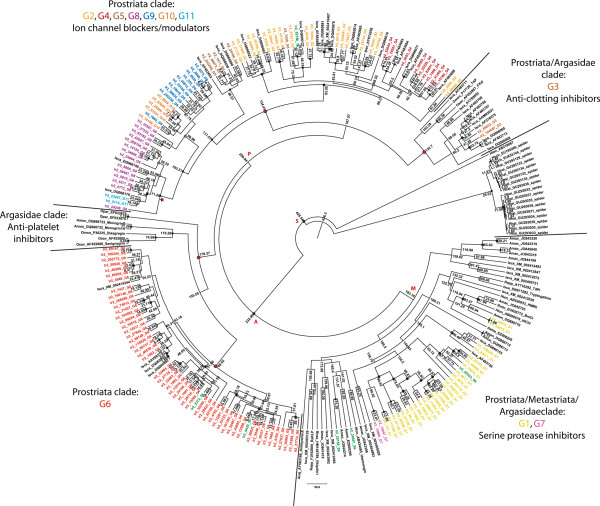
**Molecular clock analysis of *I. ricinus *Kunitz peptides.** Kunitz nucleotide sequences of *I. ricinus* groups (G1-G11 and SK) and of ticks and spiders (defined by GenBank accession numbers) were used to infer divergence times within ticks by a Bayesian uncorrelated relaxed lognormal molecular clock model. Four taxon sets were calibrated according to Jeyaprakash and Hoy [63] and the nodes with the recovered divergence times were labeled: Araneae/Scorpions/Pycnogonida/Acari 459±18 MYA (node S), Argasidae 214±28 MYA (node A), Prostriata 196±27 MYA (node P) and Metastriata 134±22 MYA (node M). The figure shows a maximum clade credibility tree including the mean divergence times (MYA) at each node. Well supported nodes and matching ML and Bayesian analyses (bootstrap values ≥70%, posterior probabilities ≥0.7, see Additional file 4) are indicated with black circles. Nodes with gray circles appeared in the ML and Bayesian trees, but they were not well supported (bootstrap values ≤70% and posterior probabilities ≤0.7). Black squares indicate well supported nodes by the Bayesian analysis (posterior probabilities ≥0.7), but were not resolved in the ML tree. Gray squares indicate well supported Bayesian tree nodes (posterior probabilities ≥0.7) that were less supported in the ML analysis (bootstrap values ≤70%). Non-labeled nodes were not well supported in both analyses (bootstrap values ≤70% and posterior probabilities ≤0.7) and they did not match in both phylogenetic reconstructions. The four best-supported clades (supported well by both analytical methods) are labeled with red circles at the nodes. The red star at the node highlights a clade containing G8 and G11 members at a divergence time of 71 MYA that is similar to the divergence time of G6. The scale bar in MYA is at the bottom center of the tree.

The phylogeny of the *I. ricinus* Kunitz peptides from our study was reconstructed and divergence times were estimated. Maximum likelihood (ML) and Bayesian phylogenetic methods resulted in similar tree topologies (Figure 6 and Additional file 4A). Four main clades containing 9 out of 11 *I. ricinus* Kunitz peptide groups were well supported in the ML (Additional file 4A: bootstrap values ≥72, marked in red) and Bayesian trees (Additional file 4B: posterior probabilities ≥ 0.7, marked in red). The largest clade out of these four clades was composed of only Kunitz peptides of prostriate ticks, namely from *I. scapularis* and *I. ricinus* groups G2, G4-5, G8-10 that we characterize as ion channel blockers/modulators. Group G11 also appeared in this clade and, regarding its transcript read profile (Figure 2C), G11 may also function as an ion channel blocker/modulator like the other Kunitz members of that clade. Although the maxiK channel modulator Ra-KLP from *R. appendiculatus* did not group with the “ion channel blocker/modulator” clade, prostriate tick salivary proteins may completely function differently than metastriate tick proteins. Thus, our characterization of G2, G4-5, G8-11 as ion channel blockers/modulators may still be valid and future experimental verifications will clarify this uncertainty.

The second largest clade also contained only prostriate Kunitz peptides composed mainly of *I. ricinus* group G6. As in the ion channel blocker/modulator clade, no functionally described Kunitz appeared in this clade, therefore any possible function remains unknown. Functionally described Kunitz peptides were present in the remaining two largest clades. One of these clades was made up of only argasid Kunitz peptides that included anti-platelet inhibitors. This group seem to evolved independently from hard ticks as previously postulated by Mans et al. [42], possibly due to different blood feeding behavior compared with hard ticks. The other clade mainly contained *I. scapularis* peptides together with *I. ricinus* group G3 and anti-clotting inhibitors of *Ornithodoros* spp.. Our aa sequence and structural analyses of G3 did not reveal any putative function for this *I. ricinus* group, although the transcript profile of G3 was similar to that of ion channel blockers/modulators (as G11 in Figure 2C). However, since G3 clusters with anti-clotting inhibitors and not with G11 may suggest a different function during tick feeding. *I. ricinus* groups G1 and G7 clustered together with hard and soft tick Kunitz peptides, but their relationships are uncertain since they were not well supported in the ML and Bayesian trees (Additional file 4). Nevertheless, in our Bayesian analysis, *I. ricinus* groups G1 and G7 appeared with functionally described serine protease inhibitors and G7 proteins were also grouped together with G1 proteins in our ML analysis. Both our phylogenetic and structural analyses of the *I. ricinus* groups G1 and G7, suggests that these two groups may act as serine protease inhibitors. SK peptides appeared throughout the phylogram. Some SKs were similar in their aa sequence (Additional file 5) with the other *I. ricinus* Kunitz peptides assuming a similar function during blood feeding. Others SKs may be more functionally distinct, such as *Ir2-24967-SK*, since it differs in its aa sequence from all other *I. ricinus* Kunitz peptides.

Our molecular clock analysis of tick and spider Kunitz peptides recovered similar divergence times for the main splits of 1) spiders from ticks (426 MYA, 95% HPD: 389–462 MYA, node S), 2) soft ticks from hard ticks (233 MYA, 95% HPD: 198–266 MYA, node A) and 3) prostriate ticks within Ixodidae (221 MYA, 95% HPD: 181–254 MYA, node P) as calibrated for these taxon sets; the tree root was estimated as 436 MYA (95% HPD: 395–479 MYA) (Figure 6, Additional file 4B). Most of these splits were well supported in the chronogram. Metastriate Kunitz peptides (fourth taxon set), however, started to evolve earlier than calibrated. Nevertheless, this split is not well supported in the chronogram as previously stated (193 MYA, 85% HPD: 160–228 MYA, Figure 3, node M). Although *I. scapularis* homologs of *I. ricinus* group G1 Kunitz peptides are considered to be the ancestral form of all Kunitz-domain proteins [23], our phylogenetic reconstructions of *I. ricinus* Kunitz peptides cannot confirm these findings. Even though G1 is not well supported in the ML and Bayesian analyses, it can be assumed that this group of proteins evolved convergently in ticks as both phylogenetic trees indicate (Additional file 4). Expansion of protein families due to gene duplication events has been previously reported for ticks [23,64] and can explain the convergent evolution of G1. Overall, the *I. ricinus* Kunitz peptides in our molecular clock analysis underlie recent gene duplication events that explains how several members of the *I. ricinus* groups and other tick Kunitz peptides do not strictly follow the calibrated divergence times within the taxon sets in the phylogenetic trees.

### *Target-oriented evolution*, a model for *I. ricinus* SG Kunitz peptide evolution

The Red Queen hypothesis [57] explains that host-parasites interactions must be characterized by constant mutations in both systems in order for parasites to efficiently infect the host and for the host to survive or avoid the parasitic attack. We understand the evolution of *I. ricinus* salivary Kunitz family in this dynamic framework. In the following paragraphs we explain our basis to propose one model of *target-oriented evolution* for this family of proteins and briefly discuss our findings regarding the evolution of venomous proteins.

Our model considers G6, compared with all other *I. ricinus* Kunitz peptide groups, as a cornerstone in understanding the Kunitz family evolution of *I. ricinus*. Firstly, members of G6 show unique molecular, structural and evolutive properties by possessing the highest amount of intra-Cys residues between Cys2 and Cys4 (Figure 1), a distinct Illumina read profile (Figure 2C), a high aa variability (Figure 3A and D), a higher number of different secondary structures (Figure 3B), and an increased number of negatively selected sites (Figure 5 and Table 1). Secondly, in the phylogenetic analysis, the clade formed by G6 is mainly composed of *I. ricinus* Kunitz peptides (Figure 6 and Additional file 4), also representing the largest monophyletic clade suggesting a recent and specific evolution for this group in *I. ricinus*. Thirdly, based on the molecular clock analysis G6 is the fastest evolving group, diverging about 70 MYA ago and having the highest number of members. Taking into account the time of divergence and the number of members, we should also consider G6 as an example of possessing an accelerated rate of evolution that has been reported for several other families of venomous proteins [5]. At this point we consider it important to make a distinction. As depicted in the phylogenetic tree (Figure 6) members of other groups appear to evolve around the same time point as G6. It is crucial to note here that even when some genes show the same time of evolutive divergence, as they belong to a distinct group, they do have different functional states at the moment of divergence. For example, our tree depicts a clade containing members of G8 and G11 that diverged 71 MYA ago (Figure 6, marked with a red star), but, as we previously showed (Figures 1, 2, 3B, 4A-B), these groups have different properties compared with G6. In this way we consider that molecular mechanisms working for the observed Kunitz family expansion (e.g., gene duplication) must be operating on the whole genome background, thus, functionally and structurally different gene(s) may show genetic amplification at similar evolutive times. But we consider that the genetic expansion must be occurring at different rates for different groups of genes, depending on the function of the gene and its importance in the interaction with the host – as previously reported for venomous protein families [62].

**Figure 7:**
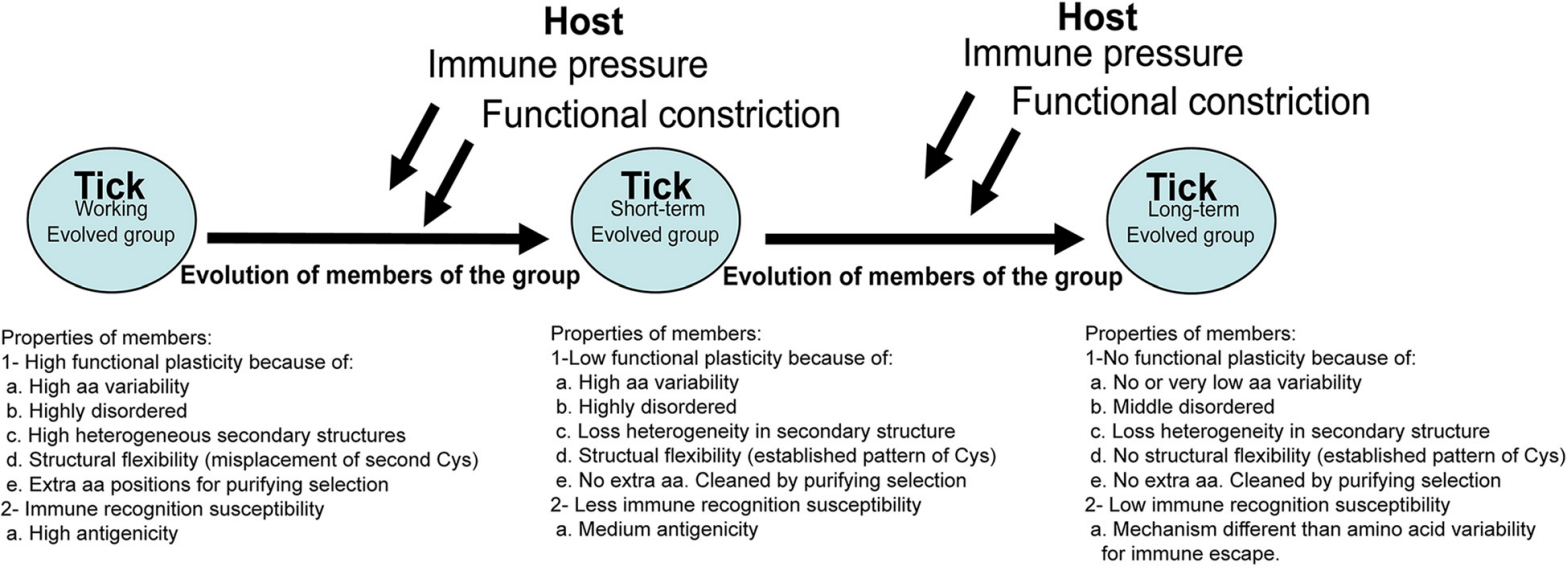
**Proposed mechanism for target-oriented evolution.** We infer from this model that aa variability, structural changes and protein disorder may have different purposes in what we label as working, short-term and long-term evolved groups of tick salivary Kunitz peptides. For working evolved groups we propose that they possess high functional plasticity, but are highly susceptible to immune recognition. In short-term evolved groups they possess low functional plasticity and less immune recognition susceptibility. Long-term evolved groups have extremely low functional plasticity and possess low immune recognition susceptibility and may possess immune escape mechanisms other than antigenic variability.

We consider that G6 is an important proof that tick salivary Kunitz peptides possess a *target-orientated evolution*. By *target-orientated evolution* we mean the molecular properties that arise in the parasitic proteins through its interactions with the host targets and the host immune system that eventually determine its structural state. Our model (Figure 7) takes into consideration that different groups are undergoing different evolutive pressures (Table 1). This may explain, from a structural point of view, the evolution of the various functions found in the Kunitz family. In the model, we propose three main evolutionary categories: “working”, “short-term” and “long-term” (Figure 7). The “working” group (i.e., G6) contains an increased number of highly variable members and will eventually reduce its number of members and gain in molecular specialization due to immunological and functional constrictions. Thus, becoming a variable but less flexible, “short-term” evolving group (i.e., G1, G2, G8 and G9). The high variability found in G1 and G2 compared with G6 may be interpreted differently. Non-monophyletic Kunitz groups, like G1 and G2 may be remnants of variable-monophyletic groups, like G6, that are evolutionarily static in expressed traits while accumulating genetic changes [65]. Finally, evolution will lead to a “long-term” evolved group (i.e., G3, G4, G5, G7, G10 and G11) that is less variable and highly specific in their molecular functions. Immune and functional pressures will remain during the whole process of both differentiation and maintenance of a gained specificity. As we showed in Table 1, *I. ricinus* SG Kunitz groups are evolving under different selective pressures and we predict they may have different functions (Figure 1 and Figure 2). Genes with different functions in venomous proteins families have been shown to evolve under different selective pressures [5] and thus at a different rate of evolution. In this sense we do not claim the three categories of “working”, “short-term” and “long-term” as relatively exclusive to time, but we also refer them to a functional state (see above the example of G6 in relation to G8 and G11).

## Conclusions

The Red Queen hypothesis is an *arms race* proposed as a model for co-evolution for host-parasite interactions [57]. Today’s increasing knowledge about the interface between host-tick interactions has provided insight that ticks successfully feed to repletion by counteracting the host immune response, platelet aggregation, inflammation and vasoconstriction [4,64,66,67]. We therefore infer that two main evolutionary pressures may be driving the evolution in *I. ricinus* Kunitz peptides – i.e., immune response and the necessity of functional diversity against the diverse host molecular targets. The former is in agreement with specific immune response against tick salivary proteins [68], and, specifically against Kunitz members [69]. The pressure for functional diversity on tick Kunitz peptides may be that several targets have been associated with their inhibitory activity such as trypsin, elastase, kallikrein [70], chymotrypsin [71], tissue factor complex inhibitor [38] and ion channels [40]. We consider our model of *target-oriented evolution* (Figure 7) to explain the evolutionary dynamics found in the *I. ricinus* SG Kunitz peptides in the framework of the Red Queen hypothesis.

## Methods

### Identifying single Kunitz-domain peptides from the *I. ricinus* transcriptome and peptide sequence alignments

We chose all full-length, secreted and non-truncated single Kunitz-domain peptide sequences from the SG *I. ricinus* transcriptome [18]. We then submitted all sequences to the Pfam server [72] to verify and identify them as single Kunitz-domain peptides. To further verify that these sequences are classical Kunitz-domain peptides we performed a protein BLAST alignment using BPTI (GenBank accession no.: P00974) as the query. As a final filtering point we submitted the remaining sequences to SignalP 4.0 server [73], since we were only interested in secreted peptides. All signal peptides were removed from all sequences and this resulted in a total of about 200 mature, single Kunitz-domain peptide sequences.

Only sequences that possessed a minimum of 6 Cys residues and a maximum of 7 Cys residues were used for our analysis. The majority of functionally described tick Kunitz peptides possess 6–7 Cys and usually >7 Cys are proteins with more than a single Kunitz-domain – with a few documented exceptions [26,40]. We specifically wanted to investigate the archetypal Kunitz-domain peptides of *I. ricinus* (hence, those possessing more or less 6 Cys residues). All peptides were divided into groups based on their Cys motif (i.e., the number intra-Cys residues) and a group was defined as whether there were two or more representative sequences for each motif. We chose a minimum of two sequences as a criterion since G3 has been previously reported by Dai et al. [23] and we only found two sequences for this group in the *I. ricinus* salivary transcriptome. This grouping resulted in a total of 145 sequences that formed eleven groups (G1-G11). We additionally found several Kunitz-domain peptides (n = 7) in the *I. ricinus* salivary transcriptome that possessed less than 6 Cys residues that were included in our phylogenetic analysis. These small peptides were defined as ‘simple Kunitz’ (SK) due their atypical Cys motif compared to archetypal Kunitz-domain peptides. Due to limitations of dealing with extremely small peptides and variations on their Cys motif, these SKs were used in only a few of our analyses. Thus, a total of 152 *I. ricinus* salivary Kunitz peptide sequences were used in our subsequent analyses.

### Tertiary protein modeling and disulfide bridge predictions

To predict the disulfide bond patterns we first modeled a representative of each group (those indicated in Figure 1) using the evolutionary protein model building web-server Phyre2 [74]. Using the Schrödinger’s Maestro software [75], each predicted 3D model was minimized, prepared, refined and re-minimized – minimization was used to remove steric clashes. Minimization uses a Truncated Newton algorithm [76], an OPLS-AA force field and a surface generalized Born (SGB) implicit solvent [77]. The total CPU time for minimizing was 2–3 min. After minimization, Cys resides were connected, hydrogen atoms were added, and the overall hydrogen-bond network was optimized using the Schrodinger’s Protein Preparation Wizard [78], which optimizes the entire hydrogen bond network by means of side chain sampling. The Protein Preparation Wizard analyzes the structure then builds a cluster of hydrogen bonds and 100000 Monte Carlo orientations for each cluster. The algorithm determines the quality of the hydrogen-bond network to produce an optimized structure. The modeled structures were then re-minimized. The disulfide bond patterns were verified using the web-server BRIDGED [79,80].

### Amino acid (aa) variability (Shannon entropy) and secondary structure analysis

Shannon entropy analysis [51] was used to calculate the aa variability. The Shannon entropy (H) was calculated for every position with the following equation:

$$H=-\sum_{i=1}^{M}P_{i}\log_{2}P_{i}$$

*P*_*i*_ is the amount of each aa type (*i*), and *M* is the number of aa types (maximum 20 aa). When *H* ≥2.0 the site is considered variable, while *H* ≤1.0 count for highly conserved positions [81]. Secondary structure was predicted using the position-specific scoring matrices method [82] from the PSIPRED server [82,83]. The presence (or not) of β-strands and α-helices, their length, amount, and position in the sequences were used as criteria for 2D model differentiation.

### Nucleotide substitution rate analysis (SNAP and Datamonkey server)

We first performed a codon-based alignment using the GUIDANCE server [84] (MAFFT, Localpair, maxiterate 1000 with 100 bootstraps) to analyze the nucleotide substitution of the Kunitz encoding transcripts. We then submitted this codon-based alignment to the SNAP server that employs the Nei and Gojobori method [85]. Nei and Gojobori used the following equations to denote the type of substitution based on the assumption that a mutation in the second nucleotide position of any codon will cause a non-synonymous substitution, but mutations at the first or third position may cause either a non-synonymous or a synonymous substitution:

$$S=\sum_{i=1}^{r}s_{i}\;N=\sum_{i=1}^{r}n_{i}$$

Where *s*_*i*_ and *n*_*i*_ are the respective sites for synonymous (*S*) and non-synonymous (*N*) substitution for the *i*th codon with the total number of codons for the sequences studied (*r*). Nei and Gojobori [85] also used the following formulas to estimate the synonymous (*d*_*S*_) and the non-synonymous (*d*_*N*_) substitutions per site by applying the Jukes-Cantor formula [86]:

dS=−34ln1−43pSdN=−34ln1−43pN

We then used *d*_*S*_ and *d*_*N*_ to identify whether a group has undergone positive (>1) or purifying selection (<1); where *p*_*S*_ and *p*_*N*_ are the synonymous differences per synonymous site and non-synonymous differences per non-synonymous site, respectively. Standard deviation (σ) and variance were also calculated by the SNAP server using the methods described by Nei and Gojobori [85].

Under the Datamonkey server [58] we used the algorithm PARRIS [60] to detect positive selection and the SLAC algorithm [59] was used to calculate the ratio *d*_*N*_/*d*_*S*_. A two-tailed extended binomial distribution set at *p-value* < *0.05* was used to assess significance of both algorithms. The SLAC and PARRIS algorithms use a neighbor-joining tree with a maximum likelihood for branch lengths and substitution rates. A few of our codon alignments contained multiple segments (i.e., recombination) as analyzed by GARD [87], therefore the base frequencies and substitution rates obtained from GARD were inferred together from the entire alignment, while branch lengths were fitted to each segment separately. Based on these analyses a global *d*_*N*_*/d*_*S*_ ratio was obtained.

The SLAC algorithm was also used to detect which nucleotide substitution site were positively or negatively selected. For each synonymous and non-synonymous nucleotide substitution site, four measurements were made: normalized expected (ES and EN) and observed numbers (NS and NN). The SLAC algorithm then calculated:

dN=NN/ENdS=NS/ES

If dN < dS a codon was negatively selected and if dN > dS a codon was positively selected.

### Phylogenetic analysis of Kunitz-domain peptides from hematophagous arthropods

All 152 *I. ricinus* Kunitz peptide sequences were used for a phylogenetic reconstruction of the Kunitz-domain family within ticks. Accordingly, we searched the NCBI database for Kunitz sequences from hard and soft ticks, and additionally spider sequences were included as outgroup. Similar to the *I. ricinus* Monolaris, only secreted and full-length Kunitz-domain proteins with up to 7 Cys residues in their aa sequence were chosen. All candidate proteins were re-confirmed as single Kunitz-domain peptides using the Pfam server [72]. Additionally, sequences of single Kunitz-domain proteins that have been functionally described were also included into the phylogenetic analysis.

A codon alignment was constructed using TranslatorX [88] and then all mature 249 single Kunitz-domain protein sequences were aligned using the program MAFFT v7 [89]. For the multiple sequence alignment we applied an iterative refinement method (L-INS-I) with the BLOSUM 62 matrix (New scoring scheme, gap opening penalty: 2.0, Offset value: 0.5). The protein alignment was then used in TranslatorX to align the corresponding Kunitz nucleotide sequences of ticks and spiders to create the final codon alignment. All unaligned, flanking sequence regions of the codon alignment were trimmed and all gaps were closed apart from gaps that were introduced by up to two sequences only (final sequence lengths: 126 bp, Additional file 5). The final alignment was analyzed using jmodeltest v2.1.2 in order to select the best-fit nucleotide substitution model for the phylogenetic reconstruction [90]. A phylogenetic tree was constructed under the maximum likelihood (ML) optimality criterion and the GTR model with a gamma distribution of substitution rate and estimated base frequencies using the program RAxML (HPC v7.2.6) [91]. Tree searching and bootstrapping were performed simultaneously (−f a, 1000 bootstrap replicates).

### Divergence time estimation

The codon alignment of coding single Kunitz-domain proteins was also used to estimate divergence times among ticks using BEAST v1.7.5 [92]. For this Bayesian analysis, the following four taxon sets and divergence times (unit: million years ago, MYA) were calibrated according to Jeyaprakash and Hoy [63] using normal prior settings: Araneae/Scorpions/Pycnogonida/Acari 459 ± 18 MYA (included taxa: all tick sequences), Argasidae 214 ± 28 MYA (included taxa: all argasid sequences), Prostriata 196 ± 27 MYA (included taxa: all prostriate sequences) and Metastriata (included taxa: all metastriate sequences) 134 ± 22 MYA. All tick Kunitz-domain sequences in the Araneae/Scorpions/Pycnogonida/Acari taxon set were enforced to be kept monophyletic. Default settings were used for all other prior and operator settings in all analyses. Similar to the ML analysis, a GTR substitution model with a gamma rate distribution across sites and estimated base frequencies were applied. Two independent Beast runs with four independent Markov-chain Monte Carlo (MCMC) chains and 100,000,000 generations were performed. The starting trees were randomly generated and all trees were sampled every 10,000 generations. Different molecular clock (uncorrelated exponential and lognormal relaxed clock) [93] and speciation models [94] (Yule process and birth-death process) were tested. A uniform prior distribution for the calculations of the mean branch rates under the uncorrelated lognormal or exponential relaxed molecular clock was set. All BEAST analyses were assessed if each parameter converges on the same posterior distribution in the MCMC runs and if an effective sample size was reached using Tracer v1.5 (http://tree.bio.ed.ac.uk/software/tracer). Additionally, the marginal likelihoods were estimated using path sampling and step stone sampling methods of each analysis in order to select the appropriate model for our analysis [95]. According to our evaluation of the different BEAST runs, the uncorrelated relaxed lognormal molecular clock model was chosen with the Yule tree prior for speciation. The two tree files from the latter BEAST analyses were combined using LogCombiner v1.7.5 from the BEAST software package using a burn-in of 10% of all sampled trees was set. Finally, TreeAnnotator v1.7.5 was used to summarize all trees in order to obtain a single maximum clade credibility tree including mean node heights.

## Abbreviations

aa: Amino acid(s); BPTI: Bovine pancreatic trypsin inhibitor; EN: Early feeding nymphs; EA: Early feeding female adults; LN: Late feeding nymphs; LA: Late feeding female adults; ML: Maxium likelihood; MYA: Million years ago; SG: Salivary glands; SK: Simple Kunitz; SNAP: Synonymous non-synonymous analysis program; V/C or AVE: Variable/conserved sites or average variability.

## Competing interests

The authors declare that they have no competing interests.

## Authors’ contributions

AS drafted the manuscript and performed and interpreted the phylogenetic analysis. ACC drafted the manuscript and performed and interpreted aa variability and secondary structure analysis. JK assisted in drafting the manuscript and in interpreting the results. JJV developed the overall concept, assisted in drafting the manuscript, organized the *I. ricinus* Kunitz sequences and Illumina data and, performed and interpreted the substitution rate, structural analysis, and bioinformatics. All authors read and approved the final manuscript.

## Authors’ information

Alexandra Schwarz and Alejandro Cabezas-Cruz joint first authorship.

Jan Kopecký and James J Valdés joint senior authorship.

## Supplementary Material

Additional file 1 **Tertiary structural representation of G6 and protein structural alignment of each group representative.** The tertiary structure of the archetypal Kunitz BPTI (A; PDB: 1BPI) and the modeled G6 representative (B; *Ir2-1983*) show the disulfide bridges (indicated by roman numerals), loops (L1 and L2), the beta-sheets (β1- β2) that form the β-hairpin, and the alpha-helices (α0 and/or α1). Both BPTI and G6 are colored from the N-terminus (blue) to the C-terminus (red). The tertiary structural alignment in Panel C depicts that the Cα protein backbone for each group representative do not drastically deviate from BPTI (color codes for each structure is presented at the far right). Click here for file

Additional file 2 **Tertiary structural deviations.** The root mean square deviation (rmsd) for each group representative and BPTI were calculated using the Protein structural alignment tool, from the Maestro molecular modeling platform [75]. Click here for file

Additional file 3 **Changes in putative secondary structure of *I. ricinus *** **Kunitz peptides.** The PSIPRED server [82,83] was used to predict the secondary structure. The different Kunitz members were grouped into 22 secondary structural models. Kunitz members shown represent prototypes for the corresponding Kunitz secondary structures. The shaded grey box is the SKs (<6 Cys residues) and the graphical legend is at the bottom right-hand corner. Click here for file

Additional file 4 **Phylograms of Kunitz peptides from *I. ricinus *** **and other tick species.** Kunitz nucleotide sequences categorized into the different groups (G1-G11) as well as the simple Kunitz sequences (SK) from the 454/Illumina SG *I. ricinus* transcriptome [18] and from different tick and spider species (outgroup) (defined by GenBank accession numbers) were used for phylogenetic reconstruction by ML and Bayesian methods. (A) In the presented ML tree all different *I. ricinus* groups are highlighted in different colors and the tree was rooted with Kunitz sequences from *Haplopelma hainanum* and *H. schmidti*. The bootstrap support values of 1,000 bootstrap replicates are displayed at all nodes and the values of the main four best-supported clades are labeled in red. The tree’s scale bar (mean aa substitution/site) is shown at the bottom center. (B) All *I.* ricinus, tick and spider Kunitz sequences were used to estimate divergence times using a Bayesian uncorrelated relaxed lognormal molecular clock model. Four taxon sets were calibrated according to Jeyaprakash and Hoy [63]: Araneae/Scorpions/Pycnogonida/Acari 459 ± 18 MYA, Argasidae 214 ± 28 MYA, Prostriata 196 ± 27 MYA and Metastriata 134 ± 22 MYA. The figure presents the Bayesian posterior probabilities and the age ranges (95% HPD, blue bars) at all nodes of the maximum clade credibility tree. The posterior probabilities of the main four best-supported clades are labeled in red. The scale bar in MYA is given at the bottom center of the tree. Click here for file

Additional file 5 **Multiple alignment of the single Kunitz-domain sequences.** A codon alignment of all 152 *I. ricinus* Kunitz nulecotide sequences and from other tick and spider sequences was constructed using TranslatorX [88]. Firstly, all mature 249 single Kunitz sequences were aligned using the program MAFFT v7 [89]. Secondly, the protein alignment was used in TranslatorX to align the corresponding Kunitz nucleotide sequences of ticks and spiders to create the final codon alignment. All unaligned, flanking sequence regions of the codon alignment were trimmed and all gaps were closed apart from gaps that were introduced by up to two sequences only (final sequence lengths: 126 bp). Click here for file

## References

[B1] Spielman A Mehlhorn H Voigt WP Armstrong PM Ticks 2001 2 Berlin: Springer

[B2] Dantas-Torres F Chomel BB Otranto D Ticks and tick-borne diseases: a one health perspective Trends Parasitol 2012 28 10 437 446 10.1016/j.pt.2012.07.003 22902521

[B3] Chmelar J Oliveira CJ Rezacova P Francischetti IMB Kovarova Z Pejler G Kopacek P Ribeiro JMC Mares M Kopecky J A tick salivary protein targets cathepsin G and chymase and inhibits host inflammation and platelet aggregation Blood 2010 117 2 736 744 20940421 10.1182/blood-2010-06-293241 PMC3031492

[B4] Francischetti IMB Sá-Nunes A Mans BJ Santos IM Ribeiro JM The role of saliva in tick feeding Front Biosci 2009 14 2051 2088 10.2741/3363 PMC2785505 19273185

[B5] Wong ESW Belov K Venom evolution through gene duplications Gene 2012 496 1 1 7 10.1016/j.gene.2012.01.009 22285376

[B6] Valenzuela JG Francischetti IM Pham VM Garfield MK Mather TN Ribeiro JM Exploring the sialome of the tick *Ixodes scapularis* J Exp Biol 2002 205 Pt 18 2843 2864 12177149 10.1242/jeb.205.18.2843

[B7] Ribeiro JM Francischetti IM Role of arthropod saliva in blood feeding: sialome and post-sialome perspectives Annu Rev Entomol 2003 48 73 88 10.1146/annurev.ento.48.060402.102812 12194906

[B8] Francischetti IM My Pham V Mans BJ Andersen JF Mather TN Lane RS Ribeiro JM The transcriptome of the salivary glands of the female western black-legged tick *Ixodes pacificus* (Acari: Ixodidae) Insect Biochem Mol Biol 2005 35 10 1142 1161 10.1016/j.ibmb.2005.05.007 16102420 PMC2887698

[B9] Ribeiro JM Alarcon-Chaidez F Francischetti IM Mans BJ Mather TN Valenzuela JG Wikel SK An annotated catalog of salivary gland transcripts from *Ixodes scapularis* ticks Insect Biochem Mol Biol 2006 36 2 111 129 10.1016/j.ibmb.2005.11.005 16431279

[B10] Alarcon-Chaidez FJ Sun J Wikel SK Transcriptome analysis of the salivary glands of *Dermacentor andersoni* Stiles (Acari: Ixodidae) Insect Biochem Mol Biol 2007 37 1 48 71 10.1016/j.ibmb.2006.10.002 17175446

[B11] Anatriello E Ribeiro J de Miranda-Santos I Brandao L Anderson J Valenzuela J Maruyama S Silva J Ferreira B An insight into the sialotranscriptome of the brown dog tick, *Rhipicephalus sanguineus* BMC Genomics 2010 11 1 450 10.1186/1471-2164-11-450 20650005 PMC3091647

[B12] Karim S Singh P Ribeiro JMC A Deep Insight into the Sialotranscriptome of the Gulf Coast Tick. *Amblyomma maculatum* PLoS ONE 2011 6 12 e28525 10.1371/journal.pone.0028525 22216098 PMC3244413

[B13] Ribeiro J Anderson J Manoukis N Meng Z Francischetti I A further insight into the sialome of the tropical bont tick, *Amblyomma variegatum* BMC Genomics 2011 12 1 136 10.1186/1471-2164-12-136 21362191 PMC3060141

[B14] Batista IFC Chudzinski-Tavassi AM Faria F Simons SM Barros-Batestti DM Labruna MB Leão LI Ho PL Junqueira-de-Azevedo ILM Expressed sequence tags (ESTs) from the salivary glands of the tick *Amblyomma cajennense* (Acari: Ixodidae) Toxicon 2008 51 5 823 834 10.1016/j.toxicon.2007.12.011 18243270

[B15] Aljamali MN Ramakrishnan VG Weng H Tucker JS Sauer JR Essenberg RC Microarray analysis of gene expression changes in feeding female and male lone star ticks, *Amblyomma americanum* (L) Arch Insect Biochem Physiol 2009 71 4 236 253 10.1002/arch.20318 19514082 PMC2740618

[B16] Francischetti IMB Anderson JM Manoukis N Pham VM Ribeiro JMC An insight into the sialotranscriptome and proteome of the coarse bontlegged tick, *Hyalomma marginatum rufipes* J Proteomics 2011 74 12 2892 2908 10.1016/j.jprot.2011.07.015 21851864 PMC3215792

[B17] Chmelar J Anderson J Mu J Jochim R Valenzuela J Kopecky J Insight into the sialome of the castor bean tick, *Ixodes ricinus* BMC Genomics 2008 9 1 233 10.1186/1471-2164-9-233 18489795 PMC2410133

[B18] Schwarz A von Reumont BM Erhart J Chagas AC Ribeiro JMC Kotsyfakis M De novo Ixodes ricinus salivary gland transcriptome analysis using two next-generation sequencing methodologies FASEB J 2013 27 12 4745 4756 10.1096/fj.13-232140 23964076 PMC3834774

[B19] Mans BJ Andersen JF Francischetti IMB Valenzuela JG Schwan TG Pham VM Garfield MK Hammer CH Ribeiro JMC Comparative sialomics between hard and soft ticks: implications for the evolution of blood-feeding behavior Insect Biochem Mol Biol 2008 38 1 42 58 10.1016/j.ibmb.2007.09.003 18070664 PMC2211429

[B20] Ribeiro JMC Labruna MB Mans BJ Maruyama SR Francischetti IMB Barizon GC de Miranda Santos IKF The sialotranscriptome of *Antricola delacruzi* female ticks is compatible with non-hematophagous behavior and an alternative source of food Insect Biochem Mol Biol 2012 42 5 332 342 10.1016/j.ibmb.2012.01.003 22306723 PMC3351099

[B21] Francischetti IMB Meng Z Mans BJ Gudderra N Hall M Veenstra TD Pham VM Kotsyfakis M Ribeiro JMC An insight into the salivary transcriptome and proteome of the soft tick and vector of epizootic bovine abortion, *Ornithodoros coriaceus* J Proteomics 2008 71 5 493 512 10.1016/j.jprot.2008.07.006 18725333 PMC2617759

[B22] Francischetti IMB Mans BJ Meng Z Gudderra N Veenstra TD Pham VM Ribeiro JMC An insight into the sialome of the soft tick, *Ornithodorus parkeri* Insect Biochem Mol Biol 2008 38 1 1 21 10.1016/j.ibmb.2007.09.009 18070662 PMC2233652

[B23] Dai S-X Zhang A-D Huang J-F Evolution, expansion and expression of the Kunitz/BPTI gene family associated with long-term blood feeding in *Ixodes scapularis* BMC Evol Biol 2012 12 1 4 10.1186/1471-2148-12-4 22244187 PMC3273431

[B24] Kunitz M Northrop JH Isolation from beef pancreas of crystalline trypsinogen, trypsin, a trypsin inhibitor, and an inhibitor-trypsin compound J Gen Physiol 1936 19 6 991 1007 10.1085/jgp.19.6.991 19872978 PMC2141477

[B25] Lima CA Torquato RJS Sasaki SD Justo GZ Tanaka AS Biochemical characterization of a Kunitz type inhibitor similar to dendrotoxins produced by *Rhipicephalus* (*Boophilus*) *microplus* (Acari: Ixodidae) hemocytes Vet Parasitol 2010 167 2ÄÄì4 279 287 19828254 10.1016/j.vetpar.2009.09.030

[B26] Paesen GC Siebold C Harlos K Peacey MF Nuttall PA Stuart DI A tick protein with a modified kunitz fold inhibits human tryptase J Mol Biol 2007 368 4 1172 1186 10.1016/j.jmb.2007.03.011 17391695

[B27] Gao X Shi L Zhou Y Cao J Zhang H Zhou J Characterization of the anticoagulant protein Rhipilin-1 from the *Rhipicephalus haemaphysaloides* tick J Insect Physiol 2011 57 2 339 343 10.1016/j.jinsphys.2010.12.001 21147114

[B28] Alim MA Islam MK Anisuzzaman Miyoshi T Hatta T Yamaji K Matsubayashi M Fujisaki K Tsuji N A hemocyte-derived Kunitz-BPTI-type chymotrypsin inhibitor, HlChI, from the ixodid tick *Haemaphysalis longicornis*, plays regulatory functions in tick blood-feeding processes Insect Biochem Mol Biol 2012 42 12 925 934 10.1016/j.ibmb.2012.09.005 23017545

[B29] Miyoshi T Tsuji N Islam MK Alim MA Hatta T Yamaji K Anisuzzaman Fujisaki K A Kunitz-type proteinase inhibitor from the midgut of the ixodid tick, *Haemaphysalis longicornis*, and its endogenous target serine proteinase Mol Biochem Parasitol 2010 170 2 112 115 10.1016/j.molbiopara.2009.12.005 20026198

[B30] Islam MK Tsuji N Miyoshi T Alim MA Huang X Hatta T Fujisaki K The kunitz-like modulatory protein haemangin is vital for hard tick blood-feeding success PLoS Pathog 2009 5 7 e1000497 10.1371/journal.ppat.1000497 19593376 PMC2701603

[B31] Batista IFC Ramos OHP Ventura JS Junqueira-de-Azevedo ILM Ho PL Chudzinski-Tavassi AM A new Factor Xa inhibitor from *Amblyomma cajennense* with a unique domain composition Arch Biochem Biophys 2010 493 2 151 156 10.1016/j.abb.2009.10.009 19853573

[B32] Valdés JJ Schwarz A Cabeza de Vaca I Calvo E Pedra JHF Guallar V Kotsyfakis M Tryptogalinin is a tick Kunitz serine protease inhibitor with a unique intrinsic disorder PLoS One 2013 8 5 e62562 10.1371/journal.pone.0062562 PMC3643938 23658744

[B33] Waxman L Smith D Arcuri KE Vlasuk GP Tick anticoagulant peptide (TAP) is a novel inhibitor of blood coagulation factor Xa Science 1990 248 4955 593 596 10.1126/science.2333510 2333510

[B34] Mans BJ Andersen JF Schwan TG Ribeiro JMC Characterization of anti-hemostatic factors in the argasid, *Argas monolakensis*: implications for the evolution of blood-feeding in the soft tick family Insect Biochem Mol Biol 2008 38 1 22 41 10.1016/j.ibmb.2007.09.002 18070663 PMC4274796

[B35] Karczewski J Endris R Connolly TM Disagregin is a fibrinogen receptor antagonist lacking the Arg-Gly-Asp sequence from the tick, *Ornithodoros moubata* J Biol Chem 1994 269 9 6702 6708 8120028

[B36] Mans BJ Louw AI Neitz AWH Savignygrin, a platelet aggregation inhibitor from the soft tick *Ornithodoros savignyi*, presents the RGD integrin recognition motif on the Kunitz-BPTI fold J Biol Chem 2002 277 24 21371 21378 10.1074/jbc.M112060200 11932256

[B37] Macedo-Ribeiro S Almeida C Calisto BM Friedrich T Mentele R Stürzebecher J Fuentes-Prior P Pereira PJB Isolation, cloning and structural characterisation of boophilin, a multifunctional Kunitz-type proteinase inhibitor from the cattle tick PLoS ONE 2008 3 2 e1624 10.1371/journal.pone.0001624 18286181 PMC2230226

[B38] Francischetti IM Valenzuela JG Andersen JF Mather TN Ribeiro JM Ixolaris, a novel recombinant tissue factor pathway inhibitor (TFPI) from the salivary gland of the tick, *Ixodes scapularis*: identification of factor X and factor Xa as scaffolds for the inhibition of factor VIIa/tissue factor complex Blood 2002 99 10 3602 3612 10.1182/blood-2001-12-0237 11986214

[B39] Francischetti IM Mather TN Ribeiro JM Penthalaris, a novel recombinant five-Kunitz tissue factor pathway inhibitor (TFPI) from the salivary gland of the tick vector of Lyme disease, *Ixodes scapularis* Thromb Haemost 2004 91 5 886 898 15116248 10.1160/TH03-11-0715

[B40] Paesen GC Siebold C Dallas ML Peers C Harlos K Nuttall PA Nunn MA Stuart DI Esnouf RM An ion-channel modulator from the saliva of the brown ear tick has a highly modified Kunitz/BPTI structure J Mol Biol 2009 389 4 734 747 10.1016/j.jmb.2009.04.045 19394347

[B41] Fry BG Roelants K Champagne DE Scheib H Tyndall JDA King GF Nevalainen TJ Norman JA Lewis RJ Norton RS The toxicogenomic multiverse: convergent recruitment of proteins into animal venoms Annu Rev Genomics Hum Genet 2009 10 1 483 511 10.1146/annurev.genom.9.081307.164356 19640225

[B42] Mans BJ Louw AI Neitz AWH Evolution of hematophagy in ticks: common origins for blood coagulation and platelet aggregation inhibitors from soft ticks of the Genus Ornithodoros Mol Biol Evol 2002 19 10 1695 1705 10.1093/oxfordjournals.molbev.a003992 12270896

[B43] Altschul SF Gish W Miller W Myers EW Lipman DJ Basic local alignment search tool J Mol Biol 1990 215 3 403 410 2231712 10.1016/S0022-2836(05)80360-2

[B44] Altschul SF Madden TL Schäffer AA Zhang J Zhang Z Miller W Lipman DJ Gapped BLAST and PSI-BLAST: a new generation of protein database search programs Nucleic Acids Res 1997 25 17 3389 3402 10.1093/nar/25.17.3389 9254694 PMC146917

[B45] Louw E van der Merwe NA Neitz AWH Maritz-Olivier C Evolution of the tissue factor pathway inhibitor-like Kunitz-domain-containing protein family in *Rhipicephalus microplus* Int J Parasitol 2013 43 1 81 94 10.1016/j.ijpara.2012.11.006 23220044

[B46] Kwong PD McDonald NQ Sigler PB Hendrickson WA Structure of beta-bungarotoxin: potassium channel binding by Kunitz modules and targeted phospholipase action Structure 1995 3 10 1109 1119 10.1016/S0969-2126(01)00246-5 8590005

[B47] Filshie BK Campbell IC Design of an insect cuticle associated with osmoregulation: the porous plates of chloride cells in a mayfly nymph Tissue Cell 1984 16 5 789 803 10.1016/0040-8166(84)90010-7 6515644

[B48] Benoit JB Yoder JA Lopez-Martinez G Elnitsky MA Lee RE Jr Denlinger DL Habitat requirements of the seabird tick, *Ixodes uriae* (Acari: Ixodidae), from the Antarctic Peninsula in relation to water balance characteristics of eggs, nonfed and engorged stages J Comp Physiol B 2007 177 2 205 215 10.1007/s00360-006-0122-7 17115223

[B49] Garcia-Boronat M Diez-Rivero CM Reinherz EL Reche PA PVS: a web server for protein sequence variability analysis tuned to facilitate conserved epitope discovery Nucleic Acids Res 2008 36 suppl 2 W35 W41 18442995 10.1093/nar/gkn211 PMC2447719

[B50] Diez-Rivero CM Reche PA Flower DDR, Davies M, Ranganathan S Discovery of conserved epitopes through sequence variability analysis Bioinformatics for immunomics 2009 3 New York City: Springer

[B51] Shannon CE The mathematical theory of communication Bell Syst Tech J 1948 27 379 423 623–656 10.1002/j.1538-7305.1948.tb01338.x

[B52] Tainer JA Getzoff ED Paterson Y Olson AJ Lerner RA The atomic mobility component of protein antigenicity Annu Rev Immunol 1985 3 501 535 10.1146/annurev.iy.03.040185.002441 2415142

[B53] Demchenko AP Recognition between flexible protein molecules: induced and assisted folding J Mol Recognit 2001 14 1 42 61 10.1002/1099-1352(200101/02)14:1<42::AID-JMR518>3.0.CO;2-8 11180561

[B54] Dunker AK Brown CJ Lawson JD Lakoucheva LM Obradovic Z Intrinsic disorder and protein function Biochemistry (Mosc) 2002 41 21 6573 6582 10.1021/bi012159+ 12022860

[B55] Doytchinova I Flower D VaxiJen: a server for prediction of protective antigens, tumour antigens and subunit vaccines BMC Bioinforma 2007 8 1 4 10.1186/1471-2105-8-4 PMC1780059 17207271

[B56] Kozlowski LP Bujnicki JM MetaDisorder: a meta-server for the prediction of intrinsic disorder in proteins BMC Bioinforma 2012 13 1 111 10.1186/1471-2105-13-111 PMC3465245 22624656

[B57] Van Valen ML A new evolutionary law Evol Theory 1973 1 1 30

[B58] Delport W Poon AFY Frost SDW Kosakovsky Pond SL Datamonkey 2010: a suite of phylogenetic analysis tools for evolutionary biology Bioinformatics 2010 26 19 2455 2457 10.1093/bioinformatics/btq429 20671151 PMC2944195

[B59] Kosakovsky Pond SL Frost SDW Not so different after all: a comparison of methods for detecting amino acid sites under selection Mol Biol Evol 2005 22 5 1208 1222 10.1093/molbev/msi105 15703242

[B60] Scheffler K Martin DP Seoighe C Robust inference of positive selection from recombining coding sequences Bioinformatics 2006 22 20 2493 2499 10.1093/bioinformatics/btl427 16895925

[B61] Koonin EV Logic of chance, the: the nature and origin of biological evolution 2011 Upper Saddle River: FT Press

[B62] Wong ESW Papenfuss AT Whittington CM Warren WC Belov K A limited role for gene duplications in the evolution of platypus venom Mol Biol Evol 2012 29 1 167 177 10.1093/molbev/msr180 21816864 PMC3663093

[B63] Jeyaprakash A Hoy M First divergence time estimate of spiders, scorpions, mites and ticks (subphylum: Chelicerata) inferred from mitochondrial phylogeny Exp Appl Acarol 2009 47 1 1 18 10.1007/s10493-008-9203-5 18931924

[B64] Mans BJ Neitz AWH Adaptation of ticks to a blood-feeding environment: evolution from a functional perspective Insect Biochem Mol Biol 2004 34 1 1 17 10.1016/j.ibmb.2003.09.002 14723893

[B65] Zander RH Evolutionary inferences from non-monophyly on molecular trees Taxon 2008 57 4 1182 1188

[B66] Andersen JF Structure and mechanism in salivary proteins from blood-feeding arthropods Toxicon 2010 56 7 1120 1129 10.1016/j.toxicon.2009.11.002 19925819 PMC2889010

[B67] Corral-Rodriguez MA Macedo-Ribeiro S Barbosa-Pereira PJ Fuentes-Prior P Tick-derived Kunitz-type inhibitors as antihemostatic factors Insect Biochem Mol Biol 2009 39 9 579 595 10.1016/j.ibmb.2009.07.003 19631744

[B68] Kovár L Tick saliva in anti-tick immunity and pathogen transmission Folia Microbiol (Praha) 2004 49 3 327 336 10.1007/BF02931051 15259776

[B69] Andreotti R Gomes A Malavazi-Piza KC Sasaki SD Sampaio CAM Tanaka AS BmTI antigens induce a bovine protective immune response against *Boophilus microplus* tick Int Immunopharmacol 2002 2 4 557 563 10.1016/S1567-5769(01)00203-X 11962734

[B70] Tanaka AS Andreotti R Gomes A Torquato RJ Sampaio MU Sampaio CA A double headed serine proteinase inhibitor-human plasma kallikrein and elastase inhibitor from *Boophilus microplus* larvae Immunopharmacology 1999 45 171 177 10.1016/S0162-3109(99)00074-0 10615008

[B71] Ascenzi P Bocedi A Bolognesi M Spallarossa A Coletta M De Cristofaro R Menegatti E The bovine basic pancreatic trypsin inhibitor (Kunitz 506 inhibitor): a milestone protein Curr Protein Pept Sci 2003 4 231 251 10.2174/1389203033487180 12769721

[B72] Bateman A Birney E Durbin R Eddy SR Howe KL Sonnhammer EL The Pfam protein families database Nucleic Acids Res 2000 28 1 263 266 10.1093/nar/28.1.263 10592242 PMC102420

[B73] Petersen TN Brunak S von Heijne G Nielsen H SignalP 4.0: discriminating signal peptides from transmembrane regions Nat Meth 2011 8 10 785 786 10.1038/nmeth.1701 21959131

[B74] Kelley LA Sternberg MJE Protein structure prediction on the web: a case study using the Phyre server Nat Protocols 2009 4 3 363 371 10.1038/nprot.2009.2 19247286

[B75] Schrödinger L Maestro, version 9.1 2010 New York, NY

[B76] Dembo RS Hoffman KL, Jackson RHF, Telgen J A primal truncated newton algorithm with application to large-scale nonlinear network optimization Computation Mathematical Programming 1987 31 Berlin Heidelberg: Springer 43 71

[B77] Still WC Tempczyk A Hawley RC Hendrickson T Semianalytical treatment of solvation for molecular mechanics and dynamics J Am Chem Soc 1990 112 16 6127 6129 10.1021/ja00172a038

[B78] Li X Jacobson MP Zhu K Zhao S Friesner RA Assignment of polar states for protein amino acid residues using an interaction cluster decomposition algorithm and its application to high resolution protein structure modeling Proteins Struct Funct Bioinf 2007 66 4 824 837 10.1002/prot.21125 17154422

[B79] Chen SW Pellequer JL Identification of functionally important residues in proteins using comparative models Curr Med Chem 2004 11 595 605 10.2174/0929867043455891 15032607

[B80] Pellequer JL Chen SW Multi-template approach to modeling engineered disulfide bonds Proteins Struct Funct Bioinf 2006 65 1 192 202 10.1002/prot.21059 16807887

[B81] Litwin S Jores R In theoretical and experimental insights into immunology 1992 Berlin: Springer-Verlag

[B82] Jones DT Protein secondary structure prediction based on position-specific scoring matrices J Mol Biol 1999 292 2 195 202 10.1006/jmbi.1999.3091 10493868

[B83] Buchan DWA Ward SM Lobley AE Nugent TCO Bryson K Jones DT Protein annotation and modelling servers at University College London Nucleic Acids Res 2010 38 suppl 2 W563 W568 20507913 10.1093/nar/gkq427 PMC2896093

[B84] Penn O Privman E Ashkenazy H Landan G Graur D Pupko T GUIDANCE: a web server for assessing alignment confidence scores Nucleic Acids Res 2010 38 suppl 2 W23 W28 20497997 10.1093/nar/gkq443 PMC2896199

[B85] Nei M Gojobori T Simple methods for estimating the numbers of synonymous and nonsynonymous nucleotide substitutions Mol Biol Evol 1986 3 5 418 426 3444411 10.1093/oxfordjournals.molbev.a040410

[B86] Jukes TH Cantor CR Munro HN Evolution of protein molecules Mammalian Protein Metabolism 1969 New York: Academic Press 21 132

[B87] Kosakovsky Pond SL Posada D Gravenor MB Woelk CH Frost SDW Automated phylogenetic detection of recombination using a genetic algorithm Mol Biol Evol 2006 23 10 1891 1901 10.1093/molbev/msl051 16818476

[B88] Abascal F Zardoya R Telford MJ TranslatorX: multiple alignment of nucleotide sequences guided by amino acid translations Nucleic Acids Res 2010 38 W7 W13 10.1093/nar/gkq291 20435676 PMC2896173

[B89] Katoh K Toh H Recent developments in the MAFFT multiple sequence alignment program Brief Bioinform 2008 9 286 298 10.1093/bib/bbn013 18372315

[B90] Darriba D Taboada GL Doallo R Posada D jModelTest 2: more models, new heuristics and parallel computing Nat Methods 2012 9 8 772 772 22847109 10.1038/nmeth.2109 PMC4594756

[B91] Stamatakis A RAxML-VI-HPC: maximum likelihood-based phylogenetic analyses with thousands of taxa and mixed models Bioinformatics 2006 22 2688 2690 10.1093/bioinformatics/btl446 16928733

[B92] Drummond AJ Suchard MA Xie D Rambaut A Bayesian phylogenetics with BEAUti and the BEAST 1.7 Mol Biol Evol 2012 29 8 1969 1973 10.1093/molbev/mss075 22367748 PMC3408070

[B93] Drummond AJ Ho SYW Phillips MJ Rambaut A Relaxed phylogenetics and dating with confidence PLoS Biol 2006 4 5 e88 10.1371/journal.pbio.0040088 16683862 PMC1395354

[B94] Gernhard T The conditioned reconstructed process J Theor Biol 2008 253 4 769 778 10.1016/j.jtbi.2008.04.005 18538793

[B95] Baele G Lemey P Bedford T Rambaut A Suchard MA Alekseyenko AV Improving the accuracy of demographic and molecular clock model comparison while accommodating phylogenetic uncertainty Mol Biol Evol 2012 29 9 2157 2167 10.1093/molbev/mss084 22403239 PMC3424409

