# Supplementary figures and images for: Understanding the evolutionary structural variability and target specificity of tick salivary Kunitz peptides using next generation transcriptome data
Source: BMC Evol Biol. 2014 Jan 7;14:4. doi: 10.1186/1471-2148-14-4 (PMC3890586; doi:10.1186/1471-2148-14-4)

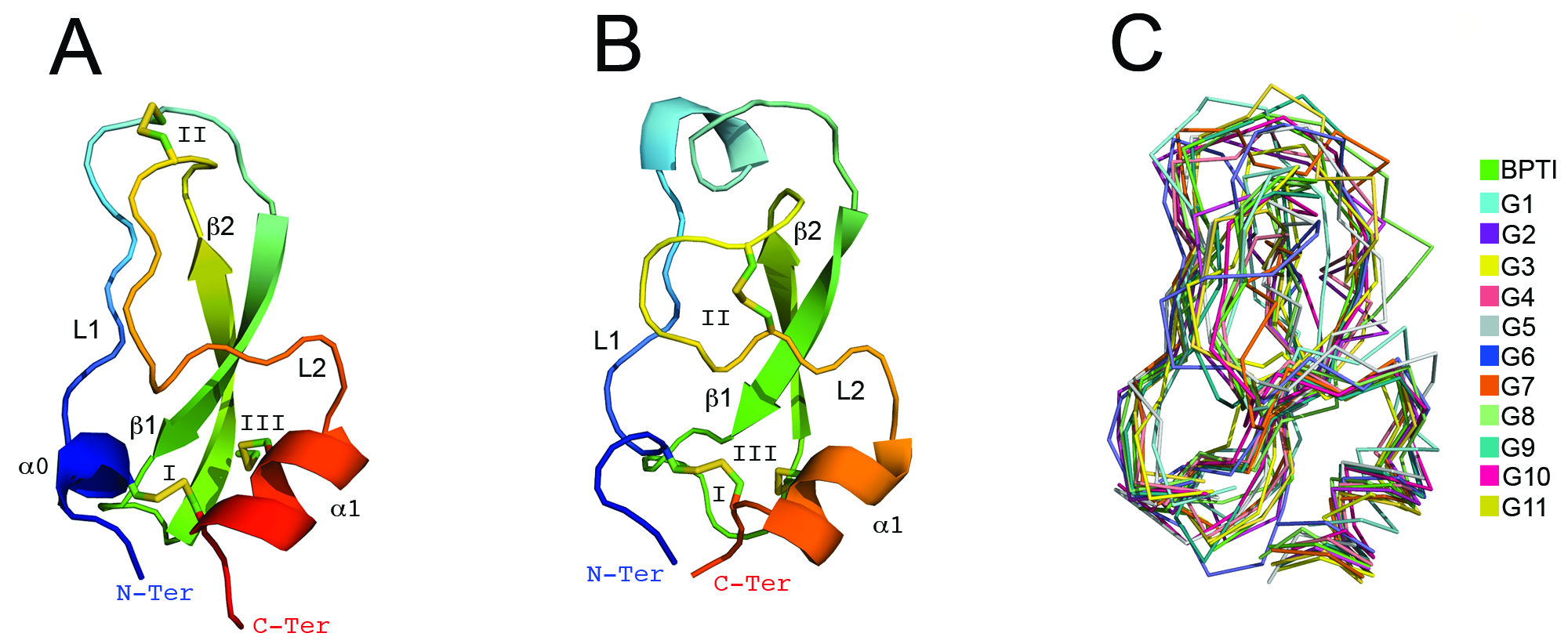

Supplement: Additional file 1 — Tertiary structural representation of G6 and protein structural alignment of each group representative. The tertiary structure of the archetypal Kunitz BPTI (A; PDB: 1BPI) and the modeled G6 representative (B; Ir2-1983) show the disulfide bridges (indicated by roman numerals), loops (L1 and L2), the beta-sheets (β1- β2) that form the β-hairpin, and the alpha-helices (α0 and/or α1). Both BPTI and G6 are colored from the N-terminus (blue) to the C-terminus (red). The tertiary structural alignment in Panel C depicts that the Cα protein backbone for each group representative do not drastically deviate from BPTI (color codes for each structure is presented at the far right). [file 1471-2148-14-4-S1.tiff]

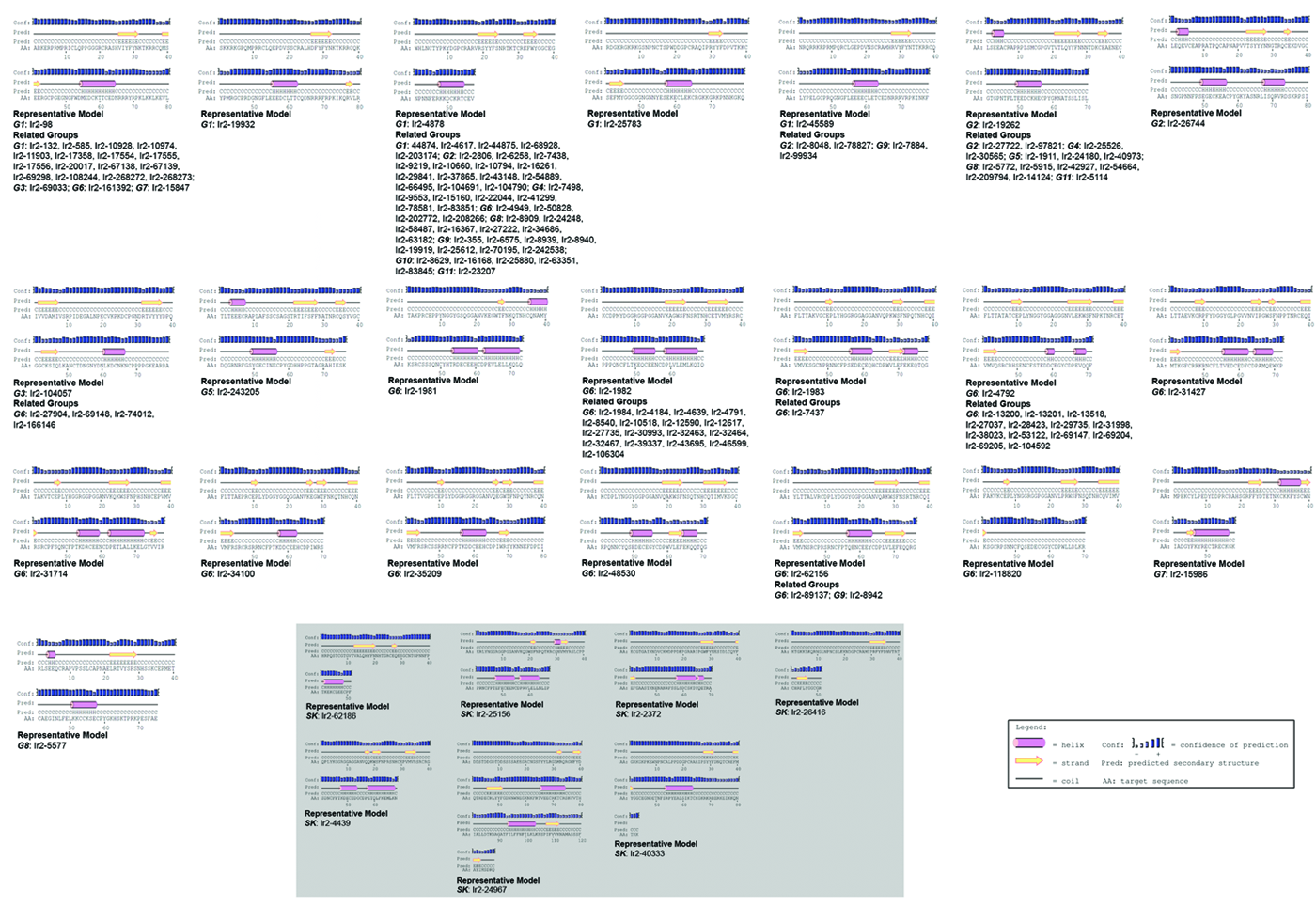

Supplement: Additional file 3 — Changes in putative secondary structure of I. ricinus Kunitz peptides. The PSIPRED server [82,83] was used to predict the secondary structure. The different Kunitz members were grouped into 22 secondary structural models. Kunitz members shown represent prototypes for the corresponding Kunitz secondary structures. The shaded grey box is the SKs (<6 Cys residues) and the graphical legend is at the bottom right-hand corner. [file 1471-2148-14-4-S3.tiff]
