# Supplementary material for: Understanding the evolutionary structural variability and target specificity of tick salivary Kunitz peptides using next generation transcriptome data
Source: BMC Evol Biol. 2014 Jan 7;14:4. doi: 10.1186/1471-2148-14-4 (PMC3890586; doi:10.1186/1471-2148-14-4)
Supplement: Additional file 4 — Phylograms of Kunitz peptides from I. ricinus and other tick species. Kunitz nucleotide sequences categorized into the different groups (G1-G11) as well as the simple Kunitz sequences (SK) from the 454/Illumina SG I. ricinus transcriptome [18] and from different tick and spider species (outgroup) (defined by GenBank accession numbers) were used for phylogenetic reconstruction by ML and Bayesian methods. (A) In the presented ML tree all different I. ricinus groups are highlighted in different colors and the tree was rooted with Kunitz sequences from Haplopelma hainanum and H. schmidti. The bootstrap support values of 1,000 bootstrap replicates are displayed at all nodes and the values of the main four best-supported clades are labeled in red. The tree’s scale bar (mean aa substitution/site) is shown at the bottom center. (B) All I. ricinus, tick and spider Kunitz sequences were used to estimate divergence times using a Bayesian uncorrelated relaxed lognormal molecular clock model. Four taxon sets were calibrated according to Jeyaprakash and Hoy [63]: Araneae/Scorpions/Pycnogonida/Acari 459 ± 18 MYA, Argasidae 214 ± 28 MYA, Prostriata 196 ± 27 MYA and Metastriata 134 ± 22 MYA. The figure presents the Bayesian posterior probabilities and the age ranges (95% HPD, blue bars) at all nodes of the maximum clade credibility tree. The posterior probabilities of the main four best-supported clades are labeled in red. The scale bar in MYA is given at the bottom center of the tree. [file 1471-2148-14-4-S4.pdf]

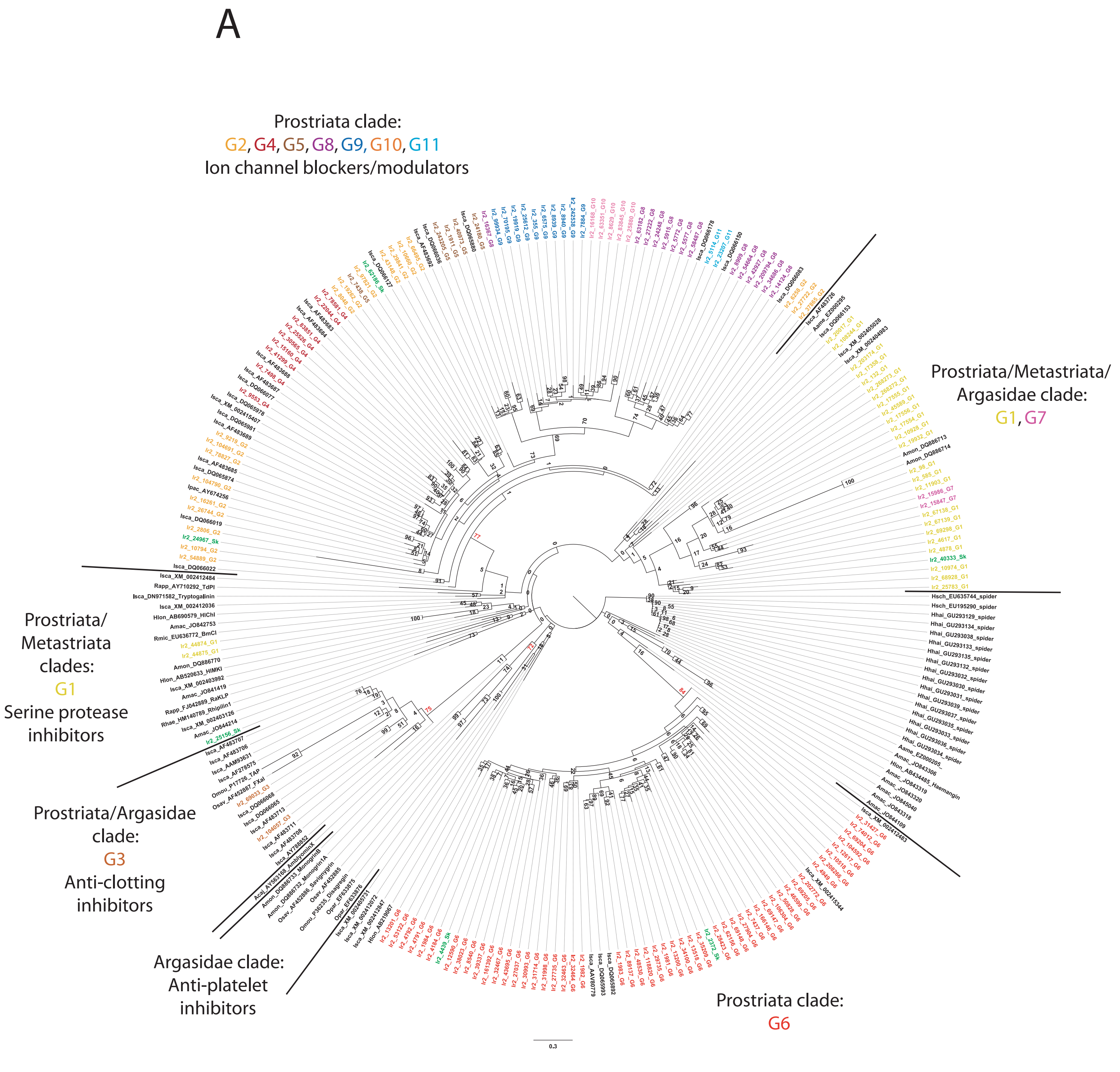
A
Prostriata clade:
G2, G4, G5, G8, G9, G10, G11
Ion channel blockers/modulators
Prostriata/Metastriata/
Argasidae clade:
G1, G7
Prostriata/
Metastriata
clades:
G1
Serine protease
inhibitors
Prostriata/Argasidae
clade:
G3
Anti-clotting
inhibitors
Argasidae clade:
Anti-platelet
inhibitors
Prostriata clade:
G6
0.3

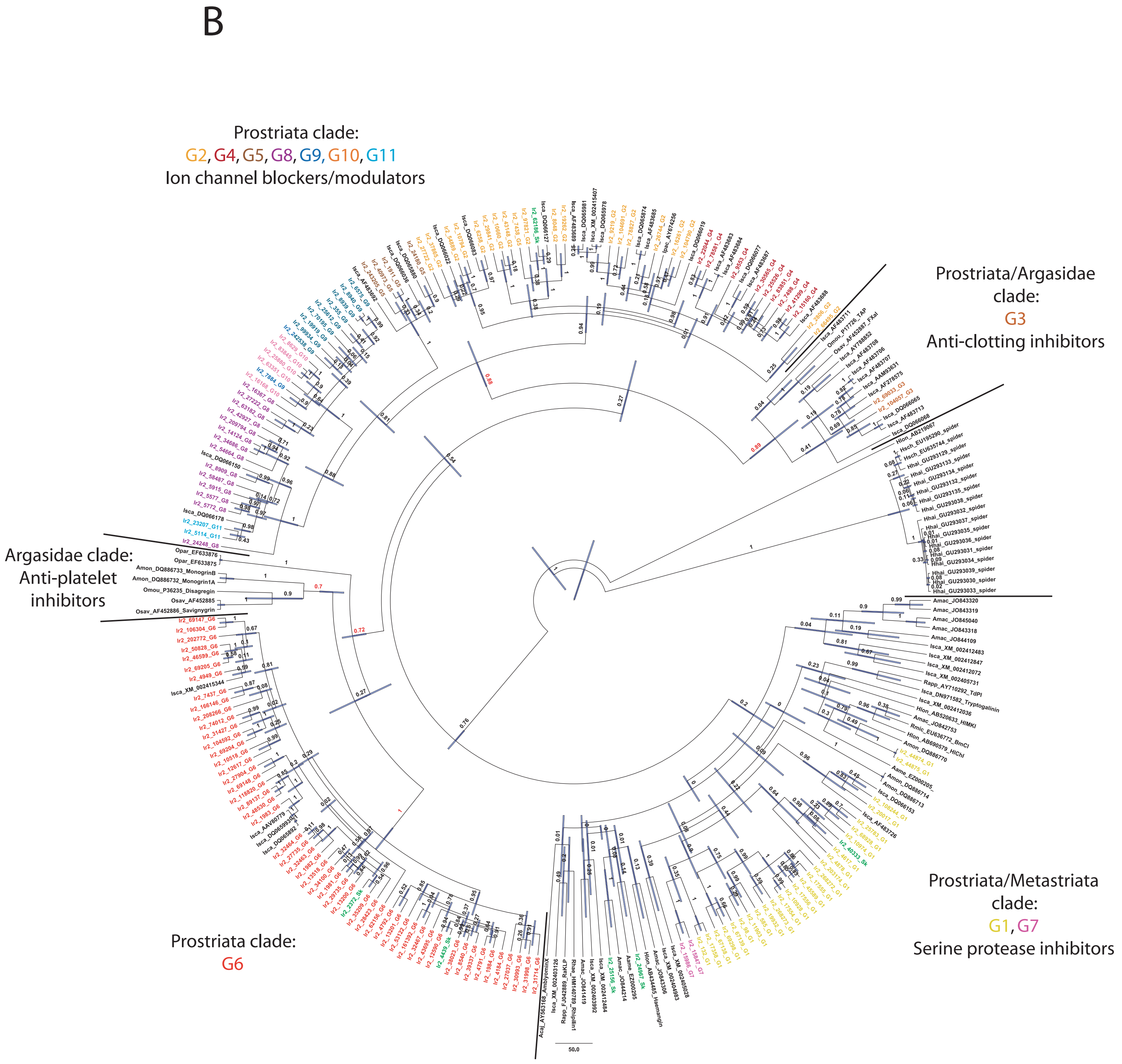
B
Prostriata clade:
G2, G4, G5, G8, G9, G10, G11
Ion channel blockers/modulators
Prostriata/Argasidae clade:
G3
Anti-clotting inhibitors
Argasidae clade:
Anti-platelet inhibitors
Prostriata/Metastriata clade:
G1, G7
Serine protease inhibitors
Prostriata clade:
G6
